# Circular RNA circACSL1 aggravated myocardial inflammation and myocardial injury by sponging miR-8055 and regulating MAPK14 expression

**DOI:** 10.1038/s41419-021-03777-7

**Published:** 2021-05-13

**Authors:** Li Zhang, Bo Han, Huanlong Liu, Jing Wang, Xinxin Feng, Wei Sun, Dongxiao Cai, Hailin Jia, Diandong Jiang

**Affiliations:** 1 Department of Pediatrics, Shandong Provincial Hospital, Cheeloo College of Medicine, Shandong University, Jinan, Shandong, 250021 China; 2 Department of Pediatrics, Shandong Provincial Hospital Affiliated to Shandong First Medical University, Jinan, Shandong, 250021 China; 3 Hand and Foot Surgery, Shandong Provincial Hospital Affiliated to Shandong First Medical University, Jinan, Shandong, 250021 China

**Keywords:** Long non-coding RNAs, Diagnostic markers, Cardiomyopathies

## Abstract

Myocarditis (MC) is a common, potentially life-threatening inflammatory disease of the myocardium. A growing body of evidence has shown that mitogen-activated protein kinase 14 (MAPK14) participates in the pathogenesis of MC. However, the upstream regulators of MAPK14 remain enigmatic. Circular RNAs (circRNAs) have been identified to play vital roles in the pathophysiology of cardiovascular diseases. Nevertheless, the clinical significance, biological function, and regulatory mechanisms of circRNAs in MC remain ﻿poorly understood. In this study, we determined a novel circRNA, circACSL1 (ID: hsa_circ_0071542), which was significantly upregulated in the acute phase of MC, and its dynamic change in expression was related to the progression of MC. We used lipopolysaccharide (LPS) to induce the inflammatory responses in the human cardiomyocytes (HCM) line for in vitro and in cellulo experiments﻿. The pro-inflammatory factors (IL-1β, IL-6, and TNF-α), myocardial injury markers (cTnT, CKMB, and BNP), cell viability, and cell apoptosis were measured to evaluate the extent of myocardial inflammation and myocardial injury level. Functional experiments, including gain-of-function and loss-of-function, were then performed to investigate the pro-inflammatory roles of circACSL1. The results revealed that circACSL1 could aggravate inflammation, myocardial injury, and apoptosis in HCM. ﻿Mechanistically, circACSL1 acted as a sponge for miR-8055-binding sites to regulate the downstream target MAPK14 expression. Furthermore, overexpression of miR-8055 rescued the pro-inflammatory effects of circACSL1 on HCM, and the upregulation of MAPK14 induced by circACSL1 was attenuated by miR-8055 overexpression. Knockdown of circACSL1 or overexpression of miR-8055 reduced myocardial inflammation and myocardial injury level and these effects were rescued by overexpression of MAPK14. In summary, our study demonstrated that circACSL1 could aggravate myocardial inflammation and myocardial injury through competitive absorption of miR-8055, thereby upregulating MAPK14 expression. Moreover, circACSL1 may represent a potential novel biomarker for the precise diagnosis of MC and offer a promising therapeutic target for MC treatment.

## Introduction

Myocarditis (MC) is a potentially life-threatening inflammatory disease of the myocardium (heart muscle) with a broad spectrum of clinical manifestations^1^. There could be several causes that lead to MC; however, the most common cause is the viral or other pathogen-mediated infectious MC^2^. The recovery period for MC greatly varies depending on accompanying medical complications, particularly for viral MC, from a couple of weeks to several months, but if the heart muscle is permanently damaged, then the patient may die from chronic cardiomyopathy even after the myocardium inflammation has subsided^3^. Fulminant myocarditis (FM) is an acute MC with the characteristics of rapidly progressing peculiar clinical presentations involving severe hemodynamic compromise and with a relatively high mortality rate^3–5^. Improving short-term and long-term prognosis is challenging but can probably be achieved by new diagnostic techniques and novel targeted therapies. Diagnosis of MC in the early stage is pretty challenging owing to its diverse clinical presentations from clinically silent to rapidly progressive heart failure, cardiogenic shock, or fatal arrhythmias^6,7^, and also because of infrequent use of an endomyocardial biopsy, the diagnostic gold standard for MC^8^. Moreover, current diagnostic tests, such as blood tests for serum cardiac biomarkers (troponin T, troponin I, creatine kinase muscle-brain isoenzyme, brain natriuretic peptide) detection, electrocardiography, echocardiography, and cardiovascular magnetic resonance are nonspecific^9^. Therefore, it is of vital importance to explore new biomarkers that can precisely diagnose the early onset of MC. The identified etiological factors are heterogeneous in nature, including viral, bacterial, or fungal pathogen-mediated infections that spread to the heart, certain immune and autoimmune condition-associated cardiac inflammation, and secondary inflammatory responses in cardiac muscles owing to drug toxicity or other illness; however, the exact pathogenesis of MC remains unclear^1,10^. Current treatment for MC can be either symptomatic or etiologic^9,11^. Therefore, elucidation of the underlying pathomechanisms of the myocardial inflammation is critical for the development of effective therapeutic strategies.

Circular RNAs (circRNAs) are a type of non-coding single-stranded RNAs with covalently closed continuous loop obtained by back-splicing of precursor mRNAs^12,13^. Without 5′–3′ polarity and polyadenylated tail, circRNAs possess high stability and can function as new biomarkers for disease diagnosis^14,15^. Studies have claimed that circRNAs exhibit vital biological functions, including transcriptional regulation, interference with splicing, binding with proteins, and translation into polypeptides, etc^14,16,17^. Of interest, circRNAs can function as competing for endogenous RNAs (ceRNAs) binding miRNAs to regulate targeted gene expression^18,19^. A growing body of evidence suggests that circRNAs play a vital role in the pathophysiology of cardiovascular diseases^20–22^, such as myocardial infarction^23^, myocardial ischemia/reperfusion injury^24,25^, myocardial fibrosis^26^, cardiomyopathy^27^, myocardial hypertrophy^28^, and heart effort failure^29^, atherosclerosis^30^, etc. However, the mechanistic roles of circRNAs in the development and progression of MC have not been explored in detail.

The mitogen-activated protein kinase (MAPK) family proteins play a pivotal role in transducing mitogenic signals from the cell surface to the nucleus in response to a wide variety of stimuli, including extracellular signal-regulated kinase (ERK1/2), p38 MAPK, and c-Jun-N terminal kinase (JNK1/2)^31^. p38 MAPK family of signaling proteins like p38α (MAPK14), p38β (MAPK11), p38γ (MAPK12), and p38δ (MAPK13), are involved in the regulation of stress-induced gene activation, cytosolic chaperone activity, cell cycle, apoptosis, cell development, proliferation, and inflammatory responses^32,33^. Among these, p38α (MAPK14), being the most abundant and well-characterized isoform, has a central role in the stress-activated initiation of pro-inflammatory responses as well as a negative regulator of proliferation of hepatocytes during acute liver injury^34^. Downstream substrates of MAPK14 signaling cascades include transcription factors and protein kinases that dictate cellular responses to stress and inflammation^35,36^. Emerging studies have shown that MAPK14 has a crucial role in the MC progression and prognosis^37–39^. Recent findings also have highlighted that MAPK14 participates in myocardial injury through activation of p38/NF-kB inflammatory signaling axis^40,41^. However, the upstream regulators of MAPK14 remain enigmatic.

Our previous investigations have provided a comprehensive expression profile of differentially expressed circRNAs, among which the expression of hsa_circ_0071542 was significantly (*p* < 0.05) upregulated in children with FM pathology^42^. This result was further validated by microarray analysis and quantitative real-time polymerase chain reaction (qRT-PCR) assay as well. In addition, the microarray data revealed that MAPK14 was upregulated in MC. Based on these intriguing findings, we hypothesized that hsa_circ_0071542 might regulate the expression of MAPK14 by binding to hsa-miR-8055 through the interactions of circRNA-miRNA-mRNA network^42^. Hsa_circ_0071542 is derived from the ACSL1 pre-mRNA transcript. Herein, we termed it as circACSL1. This study was therefore designed to elucidate whether circACSL1 was involved in the pathogenesis and progression of MC via the miR-8055/MAPK14 axis and whether it could serve as a new serum biomarker for precise and early detection of MC. Elucidation of the circACSL1-mediated underlying molecular mechanisms may lead to the development of promising therapeutic candidates for MC treatment.

## Materials and methods

### Patients and peripheral blood samples

With the informed written consent provided from the parents of each patient (<18 years old), peripheral blood samples from 22 MC subjects and 20 healthy volunteers were obtained at the Department of Pediatric Cardiology from October 2018 to August 2020. All MC cases were clinically diagnosed according to the statement of the European Society of Cardiology Working Group on Myocardial and Pericardial Diseases^9^. Control patients were healthy volunteers with age and gender matched with those of MC cases. The clinical ﻿characteristics of the MC and normal control (NC) group are presented in Table 1. In addition, to compare the expression level of circACSL1 in MC with that in dilated cardiomyopathy (DCM) subjects, 25 DCM samples were collected (the clinical characteristics of DCM are shown in supplementary file 1). ﻿All DCM cases were clinically diagnosed in strict accordance with the ﻿Scientific Statement From the American Heart Association^43^. This study was approved by the ethics committee of Shandong Provincial Hospital and was performed in accordance with the Declaration of Helsinki.

**Table 1:** Clinical characteristics of myocarditis (MC) and normal control (NC) group.

	MC group (*n* = 22)	NC group (*n* = 20)	*p* value
*Sex*			
Male	14	13	0.927
Female	8	7	
*Age*			
(years) (median, P25, P75)	8 (2.5,13.5)	8 (3.2,12.8)	0.761
*Hs-TnT*			
(mean ± SD)	1945.44 ± 1989.21	3.78 ± 0.86	***
*CKMB-M*			
(ng/ml) (mean ± SD)	57.17 ± 46.06	0.55 ± 0.37	***
*NT-Pro BNP*			
(pg/ml) (mean ± SD)	15054﻿.98 ± 12768.71	65.45 ± 50.09	***
*LVEF*			
(%) (mean ± SD)	37.59 ± 12.21	63.80 ± 0.696	***

*Hs-TnT* hypersensitive troponin T, a myocardial injury marker (normal range, 0–14 pg/ml), *CKMB-M* creatine kinase muscle-brain isoenzyme mass (normal range 0.1–4.94 pg/ml), *NT-pro BNP* NT-pro brain natriuretic peptide, an index of heart failure (normal range, 0–450 pg/ml), *LVEF* left ventricular ejection fraction, tested by echocardiography, indicating cardiac contraction ability (normal value >60%).

***p < 0.001 versus control.

### Cell culture and construction of cell inflammatory model

The human cardiomyocyte (HCM) cell line (AC16) was purchased from Fenghui Biology (Hunan, China), which was isolated from normal human ventricular tissue and immortalized to the cell line. The cell line authentication (STR Profiling Report) was shown in supplementary file 2. Cells were incubated at 37°C with 5% CO_2_ in Dulbecco’s Modified Eagle’s Medium (Invitrogen, CA, USA) supplemented with 10% fetal bovine serum (Gibco, CA, USA) and 1% penicillin-streptomycin (HyClone, UT, USA). Lipopolysaccharide (LPS) (Sigma, MO, USA) was used to induce inflammation in HCM. Passaged cells were conventionally cultured for 24 h and then starved for 12 h in a serum-free medium. Subsequently, starved cells were exposed to LPS at a dose of 10 µg/ml. The control group was treated with physiological saline instead of LPS. After culturing for 48 h, the inflammatory factors (IL-1β, IL-6, and TNF-α), myocardial injury markers (cTnT, CKMB, and BNP), cell viability, cell apoptosis, and necrosis were measured to determine whether the inflammation was successfully induced (supplementary file 3)

### Lentiviral transduction

Lentivirus vectors for overexpression of circACSL1 (LV-circACSL1), short hairpin RNA (shRNA) targeting the back-splice junction of circACSL1 (sh-circACSL1), overexpression of miR-8055 (LV-miR-8055), shRNA targeting the miR-8055 (sh-miR-8055), overexpression of MAPK14 (LV-MAPK14), and the corresponding negative controls (LV-NC, sh-NC) were designed and purchased from GeneChem, (Shanghai, China). These vectors stably express GFP reporter and harbor puromycin resistance gene cassette. Three sequences of sh-circACSL1 were selected and subsequently tested for their relative knockdown efficiency. The sequences tested are as follows: 5′-CAAAATAGACCTGAGGGTA-3′ (sh1-circACSL1), 5′-AAATAGACCTGAGGGTAGT-3′ (sh2-circACSL1) and 5′-ACCTGAGGGTAGTGGTGGT-3′ (sh3-circACSL1). The sequence of negative control shRNA(sh-NC) is 5′-TTCTCCGAACGTGTCACGT-3′. CircRNA upstream intron cyclization component (600 bp), circRNA (208 bp), and circRNA downstream intron cyclization component (600 bp) were included in the circRNA overexpression vector (LV-circACSL1). Overexpression vector sequences and detailed information of circACSL1 and mutant circACSL1 (on miR-8055-binding sites) are shown in supplementary file 4. The sequence of sh-miR-8055 is 5′-TCCGTCTGCTCATGTGCTCAAAG-3′ and its negative control sequence is 5′-TTCTCCGAACGTGTCACGT-3′. The sequence of LV-miR-8055 is 5′-CUUUGAGCACAUGAGCAGACGGA-3′. The overexpression vector sequences of MAPK14 are also shown in supplementary file 4.

According to the instructions, HCM cells were seeded at a density of 10,000 cells per well in a 24-well plate and incubated overnight. The cells were transfected with the lentiviral expression vectors at a multiplicity of infection of 20. After 24 h, the transfection mixture was replaced with a complete growth medium to avoid cell toxicity. After 72 h, the transfection efficiency was monitored using an ImageXpress Micro Confocal system and confirmed by qRT-PCR. To generate stable expression lines, the cells were selected with 5 µg/mL puromycin for 7 days. When the observed GFP expression was reached ~95%, HCM cells stably expressing sh-circACSL1, LV-circACSL1, sh-miR-8055, LV-miR-8055, LV-MAPK14, and their corresponding negative controls were used in cellulo experiments. The GFP expression captured by a fluorescence microscope is shown in supplementary file 5.

### RNA extraction and qRT-PCR analysis

Total RNA from the peripheral blood leukocytes and cultured cells was extracted using SparkZol Reagent (SparkJade, Shandong, China), as directed by the manufacturer. A NanoDrop ND-2000 spectrophotometer (NanoDrop, DE, USA) was employed to estimate total RNA quantity and quality. cDNA was synthesized using the Evo M-MLV RT Premix for qPCR (AG11706, ACCURATE BIOTECHNOLOGY, Hunan, China) and Mir-XTM miRNA First-strand Synthesis Kit (Takara, Dalian, China). RT-qPCR was conducted with SYBR Green Premix Pro Taq HS qPCR Kit (AG11701, ACCURATE BIOTECHNOLOGY, Hunan, China) as directed by the manufacturer. Primers were synthesized by BioSune Co., Ltd. (Shanghai, China) (supplementary file 6). qRT-PCR was carried out to quantitate the expression levels of circACSL1, miR-8055, and mRNAs (ACSL1, MAPK14, IL-1β, IL-6, TNF-α, cTnT, CKMB, and BNP) on a LightCycler480 system (Roche Diagnostics, Switzerland). The expression of miR-8055 was normalized to that of U6, and the others were normalized to ACTB. The relative quantification of gene expression levels was determined by the 2^−∆∆^CT method.

### CCK-8 cell proliferation assay

The normal cardiomyocytes and transfected cardiomyocytes were seeded into a 96-well plate at a density of 2000 cells/well. The cells were then treated as previously described to induce inflammation. After that, cells were incubated for 24, 48, and 72 h, and 10 µL of Cell Counting Kit (CCK)-8 solution (Dojindo, Kumamoto, Japan) was added to each well. After incubation for 2 h at 37°C, the absorbance was measured using a microplate reader (EL340, Bio-Tek Instruments, MA, USA) at 450 nm.

### Flow cytometry assay

Cell apoptosis was measured using flow cytometry using Annexin V-phycoerythrin (PE)/7-aminoactinomycin d (7AAD) kit (BD Biosciences, NJ, USA) following the manufacturer’s protocol. In brief, cells in culture media were collected by centrifugation, and then the cell pellets were washed with 1× phosphate-buffered saline three times. The collected cells were then stained with annexin V-PE/7AAD for 15 min in the dark at room temperature according to the manufacturer’s instructions. Groups of 10,000 cells were analyzed using a BD LSRFortessa^TM^ (BD Biosciences, NJ, USA).

### Enzyme-linked immunosorbent assay (ELISA)

The HCM culture supernatants with different treatments were collected by centrifugation at 1000 × *g* for 20 min and then detected immediately. Levels of IL-1β and IL-6 were determined by ELISA kit (BD Biosciences, CA, USA). TNF-α, cTnT, CKMB, and BNP were detected by using another ELISA kit (Elabscience, China) according to the manufacturer’s instructions. The absorbance values (OD) were measured at the wavelength of 450 nm by using a microplate reader (EL340, Bio-Tek Instruments, MA, USA).

### RNA fluorescent in situ hybridization (FISH)

The FISH assay was performed to detect the cellular location of circACSL1 and its colocalization with miR-8055. CY3-labeled circACSL1 probes and FAM-labeled miR-8055 probes were designed and synthesized by GenePharma (Shanghai, China). The sequence of circACSL1 probe is 5′-CCCTCAGGTCTATTTTGAGCAAAGA-3′ and the sequence of the miR-8055 probe is 5′-TCCGTCTGCTCATGTGCTCAAAG-3′. The signals of the probes were detected by RNA-FISH Kit (GenePharma, Shanghai, China) according to the manufacturer’s instructions. In brief, HCMs were seeded in a climbing piece for 24 h. After fixation and hybridization, cells were incubated with probes (4 µM) at 37°C overnight. The nuclei were stained with DAPI for 10 min. Finally, the images were acquired on a fluorescence microscope (OLYMPUS BX63, Tokyo, Japan).

### Dual-luciferase reporter assay

The sequences of circACSL1 or the 3′-UTR of MAPK14, including wild-type (circACSL1-WT, and MAPK14-WT) or mutant miR-8055-binding sites (circACSL1-Mut and MAPK14-Mut), were synthesized and inserted into luciferase reporter vectors (Genechem, Shanghai, China). The sequences are shown in Supplementary file 7. The correctly sequenced luciferase reporter plasmids were co-transfected into cells with miR-8055 overexpression plasmids or its negative control, respectively. The luciferase activity was analyzed following the manufacturer’s instructions by using a dual-luciferase assay kit (E1910; Promega, Madison, WI, USA) after 48 h of transfection. The results are presented as the relative firefly luciferase activity, which was normalized to the activity of renilla luciferase.

### RNA pull-down

Biotinylated circACSL1 probes, miR-8055 probes, and corresponding negative control probes (NC probes) were synthesized by GENERAL BIOL (Anhui, China). The sequence of the circACSL1 probe was just complemented to the back-spliced junction of circACSL1. The sequences of circACSL1 probes, miR-8055 probes, and NC probes are listed in Supplementary file 6. Pull-down assay was performed using the RNA antisense Purification Kit (Bersin Bio, Guangzhou, China). HCM cells were cross-linked with formaldehyde and lysed. The cell lysates were incubated with probes and streptavidin magnetic beads. Finally, the beads were extracted and eluted with buffer, and the RNAs were purified and then analyzed by qRT-PCR.

### Western blot

Treated HCM cells were lysed on ice using radioimmunoprecipitation assay lysis buffer (Beyotime, China) with protease inhibitor mixture (Solarbio, Beijing, China) (100:1). The protein concentrations were determined using a bicinchoninic acid protein assay kit (Solarbio, Beijing, China). Total protein (20 μg) was separated by 10% sodium dodecyl sulfate-polyacrylamide gel electrophoresis and transferred to polyvinylidene fluoride membranes. The membranes were then blocked with 5% non-fat dry milk for 2 h at room temperature and incubated at 4°C overnight with primary antibodies. Rabbit anti-MAPK14 (1:1000, Abcam, San Francisco, CA, USA) and rabbit anti-tubulin (1:1000) (﻿CST, Danvers, MA, USA) antibodies were used for probing. ﻿After incubation, membranes were washed three times with 1× TBST for 10 min and incubated with horseradish peroxidase-conjugated goat anti-rabbit IgG secondary ﻿antibody (1:1000, Proteintech, China) for 1 h at room temperature. Protein bands were detected using enhanced chemiluminescence (Amersham Imager 600).

### RNase R assay and Sanger sequencing

To confirm the stability and characteristics of circACSL1, RNase R (Geneseed Biotech, Guangzhou, China) was used for linear RNA digestion in the total RNA pool obtained from HCM cells. A total of 5 μg of RNA extracted from cells using TRIzol reagent was incubated with 20 U/μL RNase R (3 U/μg RNA) for 30 min at 37°C. The control group was treated under RNase R-free conditions. The levels of circular RNA (circACSL1) and linear RNA (ACSL1 and ACTB, respectively) were determined using a qRT-PCR assay as previously described. The amplification products of circRNAs were collected for Sanger sequencing by BioSune (Shanghai, China) to confirm the back-splice junction of circACSL1.

### Statistical analysis

Results were obtained from at least three independent, replicate experiments. Data from the present study are presented as the means ± standard deviation (SD). The two-tailed Student’s ﻿*t* test was used for comparisons between two groups, and one-way analysis of variance was used for comparisons of multiple groups. A *p* value of < 0.05 was considered to indicate statistically significant data. Statistical analyses were performed with SPSS v.24.0 software, and visualized data were obtained with Graphpad prism 8.0 software.

## Results

### The circular nature and subcellular localization of circACSL1

**Fig. 1:**
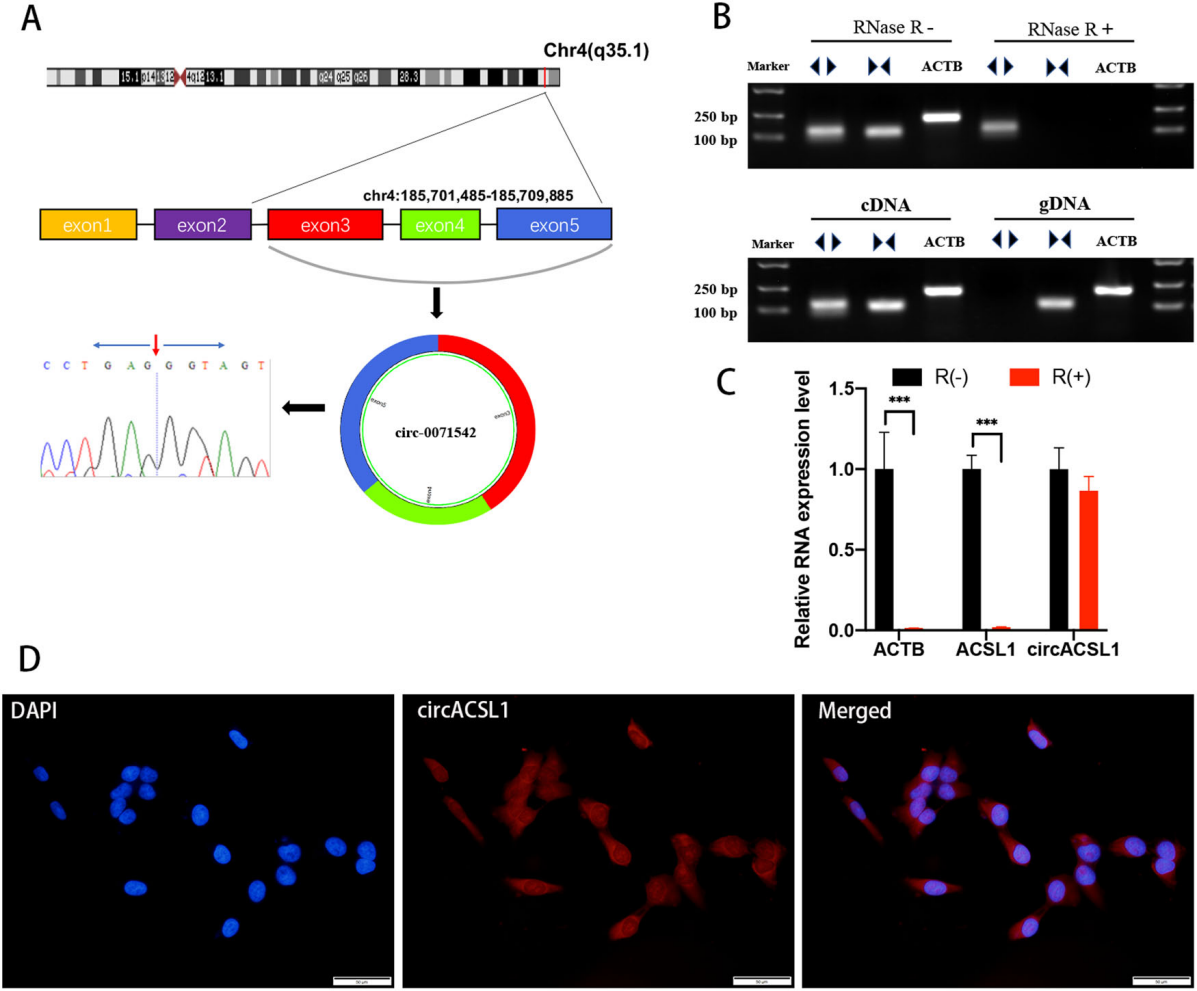
Characteristics and subcellular localization of circACSL1. **A** The genomic loci of circACSL1 (hsa_circ_0071542) and ﻿schematic representation of circACSL1 formation. The expression of circACSL1 was validated by qRT-PCR using a spanning junction primer followed by Sanger sequencing. ﻿The red arrow represents head-to-tail circACSL1 splicing sites. ﻿**B** Northern blot analysis for the detection of circACSL1 expression in HCM treated with or without RNase R; divergent primers amplified circACSL1 from cDNA, but not from gDNA. ACTB was used as a negative control. **C** The expression of circACSL1 and ACSL1 mRNA in HCM cells treated with or without RNase R was detected by qRT-PCR. The relative levels of circACSL1 and ACSL1 mRNA were normalized to the values measured in RNase R-treatment group. *p < 0.05, ﻿***p < 0.001. **D** ﻿RNA-FISH revealed the predominant localization of circACSL1 in the cytoplasm. circACSL1 probes were labeled with CY3 (red). Nuclei were stained with DAPI (blue). Scale bar, 50 µm.

CircACSL1 or hsa_circ_0071542 (chr4:185701485–185709885) is derived from exon 3, exon 4, and exon 5 of acyl-CoA synthetase long-chain family member 1 (ACSL1) pre-mRNA, which is located on chromosome 4 q35.1. We first designed spanning junction primers to amplify circACSL1. Then, the amplified product was sequenced using Sanger sequencing to validate the circularized junction of circACSL1 (Fig. 1A). Next, we designed convergent primers to amplify ACSL1 and divergent primers to amplify circACSL1 using both cDNA and genomic DNA (gDNA). CircACSL1 was amplified by divergent primers in cDNA but not in gDNA (Fig. 1B). Furthermore, RNase R digestion assay was performed to validate the stability and circular nature of circACSL1. We confirmed that circACSL1 was resistant to RNase R digestion by northern blotting (Fig. 1B) and qRT-PCR assay (Fig. 1C), whereas both linear RNA transcripts of ACSL1 and ACTB were significantly degraded compared with circACSL1 after RNase R treatment. We further detected the subcellular localization of circACSL1 in HCM cells by FISH, which revealed circACSL1 was predominantly localized in the cytoplasm (Fig. 1D).

### CircACSL1 is upregulated in MC and implicated in the progression of MC

**Fig. 2:**
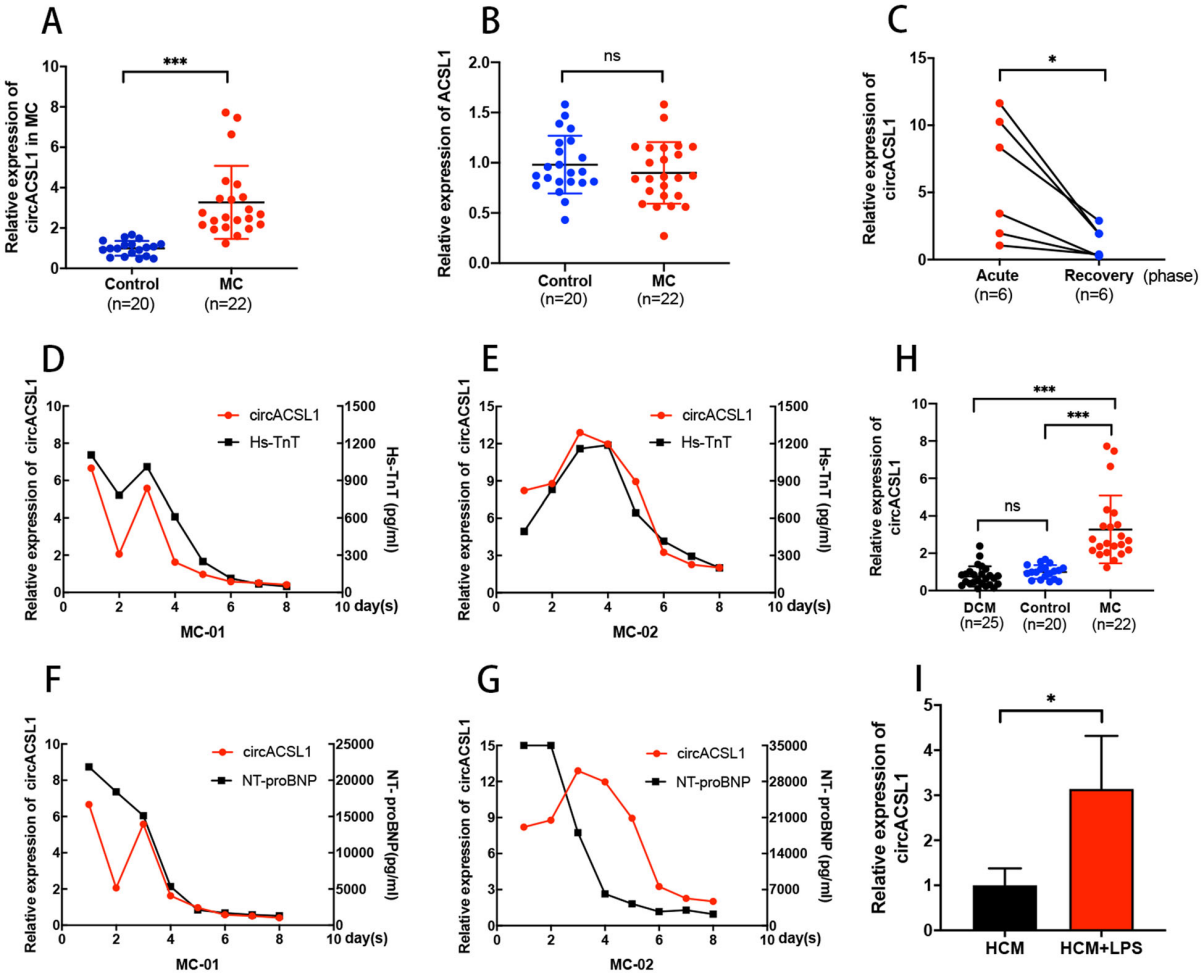
CircACSL1 is upregulated in myocarditis (MC). **A** The expression of circACSL1 was higher in MC patients (*n* = 22) compared with that in control volunteers (*n* = 20) as measured by qRT-PCR. **B** The expression of pre-mRNA ACSL1 showed no significant difference between MC (*n* = 22) and controls (*n* = 20). **C** The expression level of circACSL1 in the recovery phase decreased significantly compared with the acute phase in six MC patients (*n* = 6). **D**, **E** The dynamic changes of circACSL1 and Hs-TnT (pg/ml) levels were detected every day within the first week of onset in two MC patients (MC-01 and MC-02). **F**, **G** The dynamic changes of circACSL1 and NT-pro BNP (pg/ml) levels were detected every day within the first week of onset in two MC patients (MC-01 and MC-02). **H** The expression levels of circACSL1 in dilated cardiomyopathy (DCM) and MC. **I** circACSL1 was upregulated in the LPS-induced HCM inflammation model. ﻿Data were presented as mean ±SD. *p < 0.05; **p < 0.01; ***p < 0.001; ns (no significance) *p* > 0.05 (Student’s *t* test).

In the previous study, we detected the expression of circACSL1 in leukocytes separated from peripheral blood samples of eight MC children and eight healthy controls, and we found that circACSL1 level was upregulated in MC cases versus control patients. Here, we further analyzed the expression of circACSL1 in a larger sample size (22 MC samples and 20 control samples, age, and sex-matched). Consistent with previous results, the expression levels of circACSL1 in MC samples were significantly higher than those in control samples (Fig. 2A), whereas the expression level of ACSL1 pre-mRNA showed no significant difference (Fig. 2B). To explore the diagnostic value of circACSL1 as a biomarker for MC, acute phase, and recovery phase peripheral blood samples of six MC patients were collected to perform qRT-PCR assays. The results manifested that the expression of circACSL1 in the recovery phase was decreased significantly compared with that of the acute phase (Fig. 2C). Furthermore, the expression levels of circACSL1, hypersensitive troponin T (Hs-TnT), and NT-pro brain natriuretic peptide (NT-pro BNP) from two MC patients (MC-01 and MC-02) blood samples were consistently detected on a daily basis within the first week of disease onset. The results showed that the dynamic trend of circACSL1 level was consistent ﻿﻿with Hs-TnT and NT-pro BNP expression levels (Fig. 2D–G), indicating that circACSL1 might be a potential biomarker for the diagnosis of MC pathogenesis. Considering that the differential diagnosis of MC and DCM is a bit challenging in clinical investigations, we compared the expression levels of circACSL1 between 22 MC patients and 26 DCM patients to evaluate its potential diagnostic value in effectively distinguishing MC patients from DCM individuals. ﻿The results showed that circACSL1 expression in MC was dramatically higher than that in DCM and control samples, and there was no significant difference between DCM and controls (Fig. 2H). Taken together, these results suggest that circACSL1 might be a potential novel biomarker for the diagnosis and prognostic assessment of MC.

### CircACSL1 aggravates inflammation, myocardial injury, and apoptosis in HCM

**Fig. 3:**
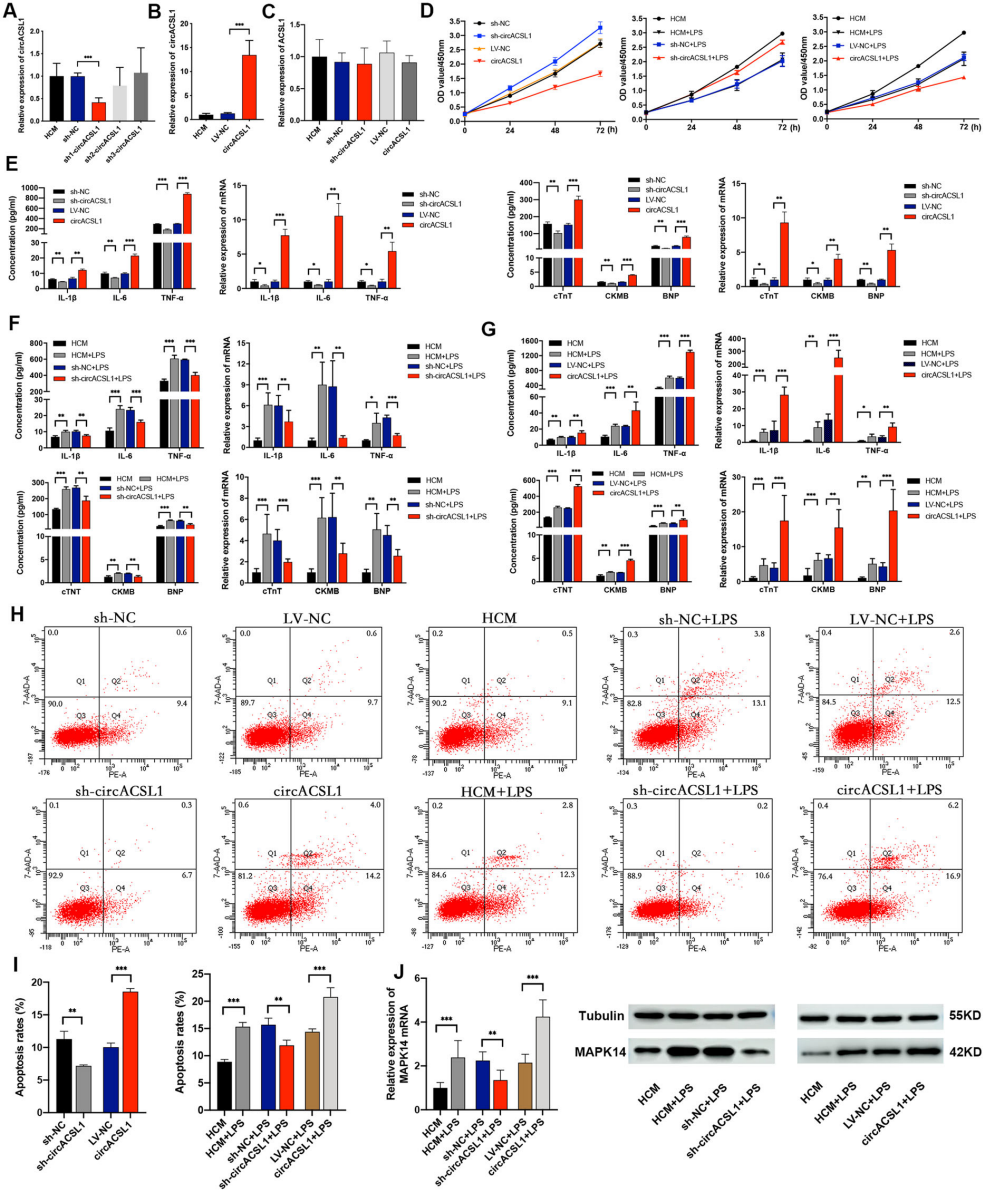
CircACSL1 aggravates inflammation, myocardial injury, and apoptosis in HCM. **A**, **B** The efficiency of knockdown or overexpression of circACSL1 in HCM cells was detected by qRT-PCR. **C** The mRNA level of ACSL1 pre-mRNA in HCM transfected with circACSL1 overexpression vector or sh-circACSL1 showed no significant differences compared with their corresponding negative controls. **D** ﻿After transfection with sh-circACSL1 or circACSL1 overexpression vector, growth curves of HCM with or without LPS stimulation were measured by the CCK-8 assay. **E**, **G** ELISA and qRT-PCR assay showed the expression levels of IL-1β, IL-6, TNF-α, cTnT, CKMB, and BNP in HCM with circACSL1 knockdown or overexpression. **E**, cells without LPS stimulation; **F**, **G**, cells with LPS stimulation. **H**, **I** ﻿Cell apoptosis was measured with annexin V-FITC/7AAD double staining using ﻿flow cytometry assay. **J** The mRNA level and protein level of MAPK14 in HCM with circACSL1 knockdown or overexpression were detected by qRT-PCR and WB, respectively. ﻿Data are presented as mean ±SD (*n* = 3 biologically independent samples). *p < 0.05; **p < 0.01; ***p < 0.001 (Student’s *t* test).

The increase of circACSL1 level in MC led us to consider whether it was related to HCM inflammation. Furthermore, to investigate the roles of circACSL1 in MC pathogenesis, we induced HCM inflammation by exposing cells to LPS to detect the expression of circACSL1, and subsequently found that the expression of circACSL1 was dramatically upregulated in LPS-induced HCM inflammation model compared with mock-treated HCM (Fig. 2I). We then speculated that circACSL1 might aggravate inflammation in HCM, so we performed loss-of-function and gain-of-function experiments to investigate the biological roles of circACSL1 in modulating inflammatory signaling cascades. Three shRNAs specifically targeting the back-splice region of circACSL1 (sh-circACSL1) were utilized to knockdown circACSL1 expression in HCM. Compared with treatment with NC, sh1-circACSL1 could significantly decrease circACSL1 level with the highest knockdown efficiency in comparison with two other shRNAs (Fig. 3A), hence, sh1-circACSL1 was used for subsequent experiments (In subsequent experiments, we called it sh-circACSL1). The circACSL1 overexpression lentivirus vector (LV-circACSL1) was transfected into HCM (empty vector as control), which exhibited ~13-fold increase in circACSL1 level (Fig. 3B). We also observed that either overexpression or knockdown of circACSL1 had no influence on the ACSL1 pre-mRNA level (Fig. 3C). The following experiments were conducted in HCM with or without LPS stimulation two conditions. CCK-8 assay indicated that circACSL1 overexpression could suppress cell viability and proliferation of HCM when exposed to LPS or not, whereas sh-circACSL1-mediated knockdown of circACSL1 expression promoted cell proliferation compared with that of NC-treated cells (Fig. 3D). qRT-PCR assay and ELISA showed that knockdown of circACSL1 resulted in the downregulation of expressions of IL-1β, IL-6, TNF-α, cTnT, CKMB, and BNP both in transcript and protein levels, whereas overexpression of circACSL1 induced the high-expression of these inflammatory cytokines (IL-1β, IL-6, and TNF-α) as well as myocardial injury biomarkers (cTnT, CKMB, and BNP) significantly, no matter HCM was exposed to LPS or not (Fig. 3E–G). We next investigated the effects of both circACSL1 inhibition and overexpression on HCM apoptosis by flow cytometry. As shown in Fig. 3H and Fig. 3I, the apoptosis rates were higher when circACSL1 was overexpressed compared with that of NC samples. As expected, knockdown of circACSL1 expression could significantly suppress apoptosis of HCM when exposed to LPS or not. In addition, sh-circACSL1-mediated circACSL1 downregulation exhibited a reduction in the MAPK14 expression level, and while conversely circACSL1 overexpression upregulated the expression of MAPK14, as observed in qRT-PCR assay and western blot (WB) results (Fig. 3J). Taken together, these results clearly indicate that circACSL1 overexpression could aggravate inflammation, myocardial injury, and apoptosis in HCM.

### CircACSL1 serves as a sponge for miR-8055 regulating the MAPK14 expression

**Fig. 4:**
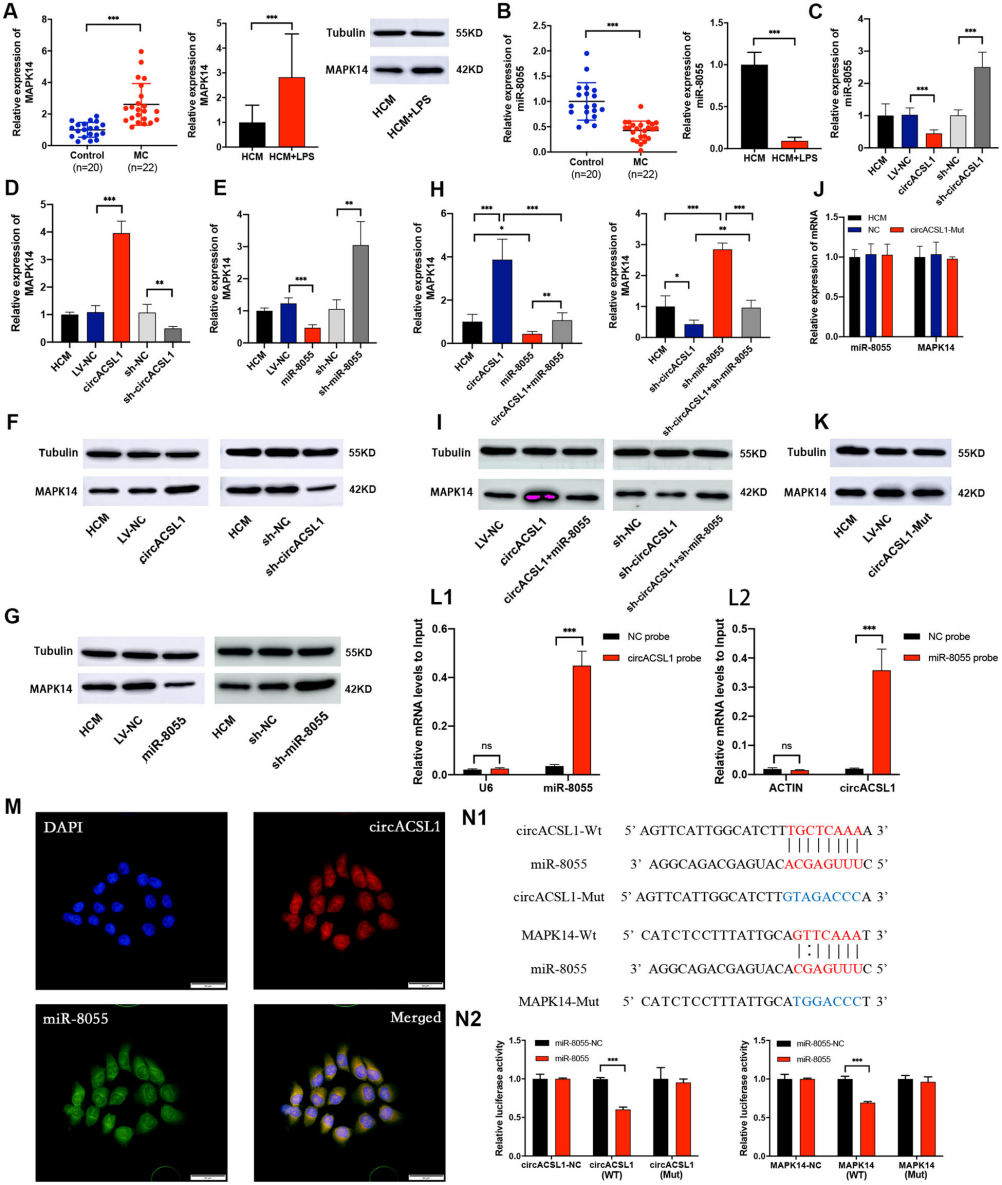
CircACSL1 serves as a sponge of miR-8055 to regulate the expression of MAPK14. **A** The expression level of MAPK14 was upregulated in MC patient samples and LPS-induced HCM inflammatory model as estimated by qRT-PCR and WB. **B** The expression level of miR-8055 was downregulated in MC patient samples and HCM inflammatory model as detected by qRT-PCR. **C** After transfection with sh-circACSL1 or circACSL1 overexpression vector, the miR-8055 expression was measured by qRT-PCR. **D** The mRNA level of MAPK14 in HCM with overexpression or knockdown of circACSL1. **E** The mRNA level of MAPK14 in HCM with miR-8055 overexpression or knockdown. **F** The protein level of MAPK14 in HCM with overexpression or knockdown of circACSL1. **G** The protein level of MAPK14 in HCM with miR-8055 overexpression or knockdown. **H**, **I** The higher expression of MAPK14 in HCM with circACSL1 overexpression could be rescued by miR-8055 overexpression, and sh-miR-8055 could relieve the inhibition of sh-circACSL1 on MAPK14 expression. **J**, **K** The expression of miR-8055 and MAPK14 could not be affected by mutant circACSL1. **L1** miR-8055﻿ was pulled down by the biotin-circACSL1 probe, detected by qRT-PCR. **L2** circACSL1 was pulled down by the biotin-miR-8055 ﻿probe, detected by qRT-PCR. The relative level was normalized to input. **M** RNA-FISH revealed the cytosolic colocalization of circACSL1 and miR-8055 in HCM. CircACSL1 probes were labeled with CY3 (red). miR-8055 probes were labeled with FAM (green). Nuclei were stained with DAPI (blue). Scale bar, 50 µm. **N1** The complementary binding sites in wild-type (WT) and mutant sites (Mut) 3′-UTR of MAPK14 and circACSL1 for miR-8055 targeting. **N2** ﻿﻿Luciferase reporter assay verified the molecular interactions of miR-8055 with the 3′-UTR of MAPK14 and circACSL1 wild-type transcripts. Data are presented as mean ±SD (*n* = 3 biologically independent samples). *p < 0.05; **p < 0.01; ***p < 0.001 (Student’s *t* test).

So far, our results have suggested that circACSL1 overexpression could exert pro-inflammatory effects on HCM cells. In order to obtain the mechanistic insights, we further explored the potential underlying mechanism of circACSL1-associated regulation of MC progression. As documented in earlier reports, exonic circRNAs, which are mainly enriched in the cytoplasm, could function as an efficient miRNA sponge by blocking the respective miRNA seed region-specific sequences on the target genes^14,19^. Fluorescent in situ hybridization targeting ribonucleic acid molecules (RNA-FISH) revealed that circACSL1 is mainly abundant in the cytoplasm (Fig. 1D). In our previous study^42^, the mRNA microarray data have shown that MAPK14 was highly expressed in MC relative to control samples, and this result was recapitulated in the current study by detecting the expression profiles of MAPK14 in MC patients samples and LPS-induced HCM inflammatory cell model (Fig. 4A). Hence, we speculated that circACSL1 might upregulate MAPK14 expression at the posttranscriptional level, most likely through a ceRNA-binding mechanism. We used online tools and RNA databases, such as miRanda, RegRNA2.0, and RNAhybrid, to predict miRNA sequences that might bind to both circACSL1 and MAPK14 mRNA transcripts. Finally, miR-8055 was screened down to have potential binding sites for circACSL1 and the 3′-UTR of MAPK14 mRNA. Then, we found that miR-8055 had a significantly low expression profile in MC patients’ samples as well as in LPS-induced HCM cells compared with controls (Fig. 4B). Subsequently, we noticed that overexpression of circACSL1 in HCM could inhibit miR-8055 maturation and expression, whereas the knockdown of circACSL1 led to upregulation of miR-8055 (Fig. 4C). qRT-PCR assay and WB results showed that the expression of MAPK14 was also significantly reduced after transfection of sh-circACSL1 vector in HCM, and on the other hand, MAPK14 expression was dramatically increased after the transfection of circACSL1 overexpression vector (Fig. 4D, F). Moreover, direct knockdown miR-8055 increased the MAPK14 expression and vice versa as shown in Figs. 4E and 4G. Furthermore, the higher expression of MAPK14 induced by circACSL1 overexpression could be rescued by miR-8055 overexpression in HCM. Consistently, sh-miR-8055 could relieve the inhibitory effects of sh-circACSL1 on MAPK14 expression (Fig. 4H, I).

We designed mutant circACSL1 sequences (circACSL1-Mut) on miR-8055-binding sites, and we found that the expression of miR-8055 and MAPK14 could not be affected by overexpression of mutant circACSL1 (Fig. 4J, K). To verify whether circACSL1 could physically interact with miR-8055, RNA pull-down assay was performed in HCM cells. The results showed that miR-8055 was significantly pulled down by the bio-circACSL1 probes, and circACSL1 was obviously enriched in the bio-miR-8055 group compared with the bio-NC group (Fig. 4L1, 4L2). Notably, RNA-FISH analysis showed that circACSL1 and miR-8055 were co-localized in the cytoplasm (Fig. 4M). To ﻿further validate the miR-8055-binding sites on the target RNAs, fragments of circACSL1 or the 3′-UTR region of MAPK14 mRNA, including the respective full-length wild-type (circACSL1-WT, MAPK14-WT) transcripts or mutant miR-8055 binding sites carrying (circACSL1-Mut, MAPK14-Mut) transcripts were synthesized and inserted into luciferase reporter vector (Fig. 4N1). ﻿Compared with NC, miR-8055 mimics significantly reduced the activity of circACSL1-WT or MAPK14-WT-fused luciferase reporter, while the circACSL1-Mut or MAPK14-Mut-fused luciferase reporter showed no significant changes in their activities (Fig. 4N2). Taken together, ﻿these results indicated that miR-8055 could bind to the corresponding sites on circACSL1 and MAPK14 mRNA transcripts and circACSL1 could regulate the expression of MAPK14 by acting as a sponge for miR-8055-binding sites.

### miR-8055 alleviates myocardial inflammation and myocardial injury in HCM

**Fig. 5:**
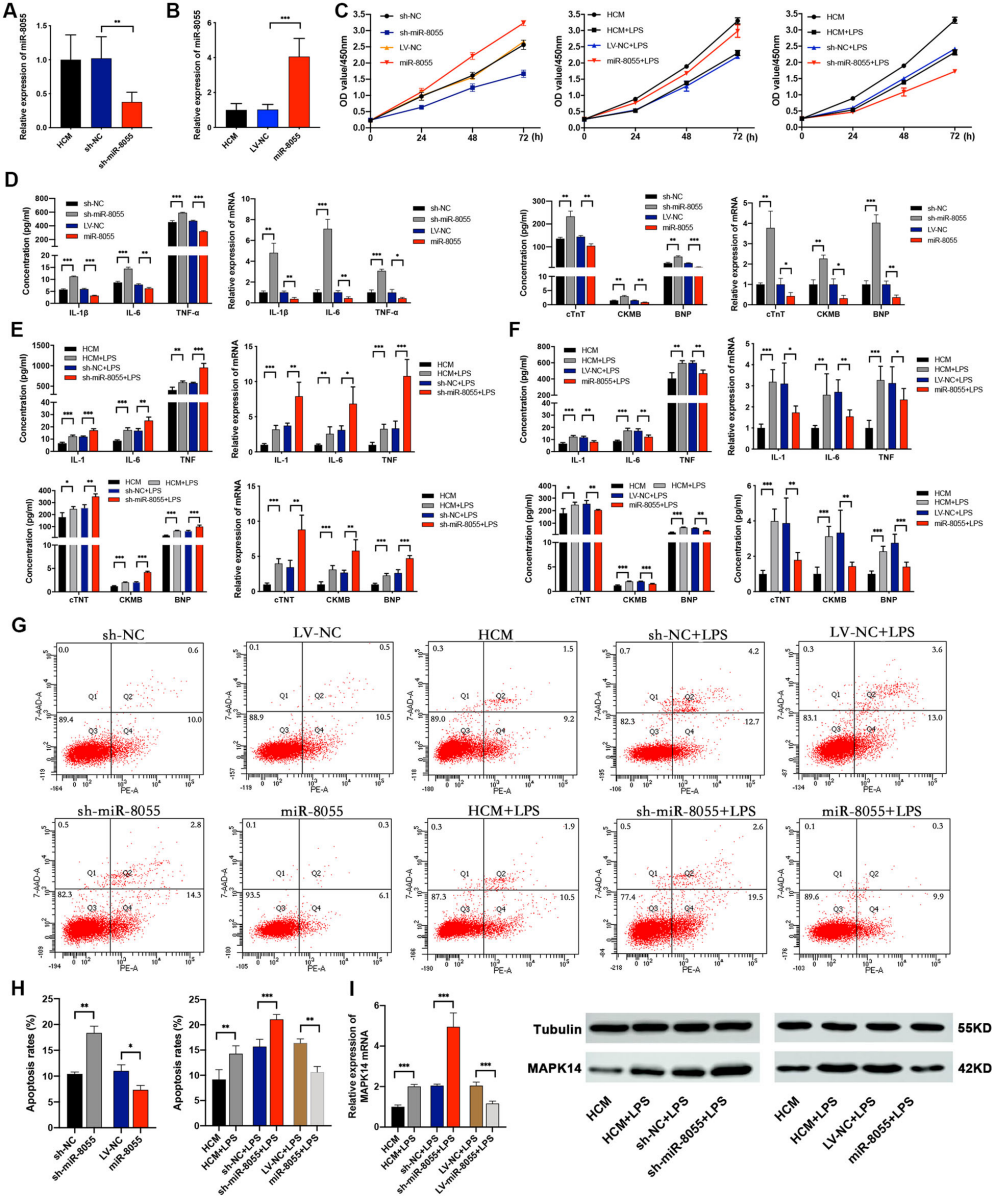
miR-8055 alleviates inflammation, myocardial injury, and apoptosis in HCM. **A**, **B** The knockdown or overexpression efficiency of miR-8055 in HCM cells was detected by qRT-PCR. **C** Proliferation curves were measured by the CCK-8 assay ﻿after knockdown or overexpression of miR-8055 in HCM cells with or without LPS stimulation. **D**–**F** The expression levels of IL-1β, IL-6, TNF-α, cTnT, CKMB, and BNP in HCM with miR-8055 knockdown or overexpression were detected by qRT-PCR and ELISA. **D**, cells without LPS stimulation; **E**, **F**, cells with LPS stimulation. **G**, **H** ﻿Cell apoptosis was detected with annexin V-FITC/7AAD double staining using ﻿flow cytometry apoptosis assay. **I** qRT-PCR and WB results showed the mRNA and protein levels of MAPK14 in HCM with miR-8055 knockdown or overexpression. ﻿Data are presented as mean ±SD (*n* = 3 biologically independent samples). *p < 0.05; **p < 0.01; ***p < 0.001 (Student’s *t* test).

To better understand the role of miR-8055 in MC, we constructed an overexpression viral vector (LV-miR-8055) to express miR-8055 and shRNA (sh-miR-8055) to knockdown miR-8055. Using qRT-PCR assay, we observed 38% knockdown efficiency of sh-miR-8055 and 4 folds of overexpression efficiency of LV-miR-8055 (Fig. 5A, B). We investigated the effects of LV- miR-8055 or sh-miR-8055 on HCM, with or without LPS stimulation. miR-8055 knockdown enhanced the expressions of IL-1β, IL-6, TNF-α, cTnT, CKMB, and BNP both in mRNA and protein levels (Fig. 5D, E), which in turn increased the apoptotic cell populations as measured by flow cytometry (Fig. 5G, H), and inhibited HCM proliferation and viability rates, as determined by CCK-8 assay (Fig. 5C). On the contrary, miR-8055 overexpression exerted opposite effects by lowering the expression of inflammatory cytokines (IL-1β, IL-6, and TNF-α) and myocardial injury biomarkers (cTnT, CKMB, and BNP) in HCM as measured by qRT-PCR and ELISA (Fig. 5D, F). Moreover, overexpression of miR-8055 also decreased the apoptotic rates and promoted HCM proliferation and viability (Fig. 5C, G, H). qRT-PCR and WB results showed that under LPS stimulation, MAPK14 was upregulated both in mRNA and protein levels with knockdown of miR-8055, and vice versa (Fig. 5I).

### CircACSL1 exerts pro-inflammatory effects and regulates MAPK14 expression by sponging miR-8055

**Fig. 6:**
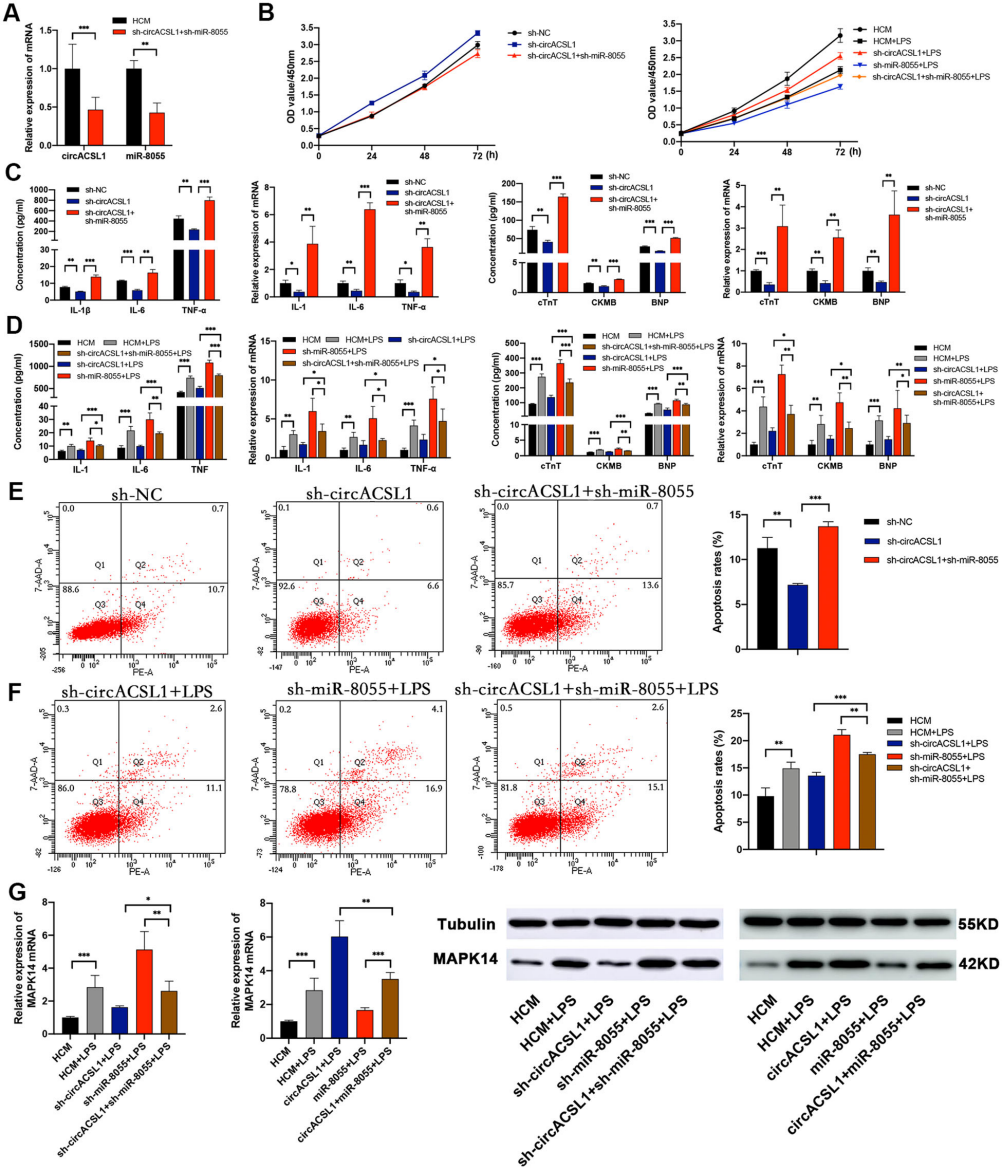
Kockdown of circACSL1-mediated attenuation in myocardial inflammation and myocardial injury can be rescued by sh-miR-8055. **A**–**G** HCM cells were co-transfected with sh-circACSL1 and sh-miR-8055, which were compared with sh-circACSL1 transfection alone or sh-miR-8055 transfection alone. **A** The knockdown efficiencies of circACSL1 and miR-8055 in HCM cells were detected by qRT-PCR. **B** Sh-miR-8055 abolished the suppressive effects of sh-circACSL1 on HCM cell proliferation. **C**, **D** Sh-circACSL1-induced downregulation of IL-1β, IL-6, TNF-α, cTnT, CKMB, and BNP were rescued by co-transfection with sh-circACSL1 and sh-miR-8055. **E**, **F** Sh-circACSL1-induced decrease of cell apoptosis was reversed by co-transfection with sh-circACSL1 and sh-miR-8055. **G** qRT-PCR and WB results showed the mRNA and protein levels of MAPK14. ﻿Data are presented as mean ±SD (*n* = 3 biologically independent samples). *p < 0.05; **p < 0.01; ***p < 0.001 (Student’s *t* test).

Based on the above results, we concluded that both circACSL1 and miR-8055 could possess biological effects on myocardial inflammation and injury, and circACSL1 could act as a sponge for miR-8055 to relieve its inhibitory effects on the regulation of MAPK14 mRNA level. MAPK14 (p38α), as an important kinase, has been found to participate in myocardial inflammation and injury in the previous studies^38,40,41^. Taken together, we speculated that cirACSL1 exerted pro-inflammatory effects in MC by sponging miR-8055 and regulating MAPK14 expression. To validate this speculation, we performed rescue experiments. HCM cells were co-transfected with circACSL1 and miR-8055 overexpression vectors, which were compared with circACSL1 overexpression alone or miR-8055 overexpression alone. Furthermore, HCM cells were co-transfected with sh-circACSL1 and sh-miR-8055, which were compared with sh-circACSL1 transfection alone or sh-miR-8055 transfection alone. The knockdown efficiencies of sh-circACSL1 and sh-miR-8055 in HCM cells were shown in Fig. 6A. The CCK-8 assay showed that the cell viability of HCM was evidently increased after knockdown of circACSL1, and this result could be reversed by miR-8055 inhibitor (Fig. 6B). Also, sh-circACSL1-mediated knockdown of circACSL1 showed a significant reduction in the expressions of IL-1β, IL-6, TNF-α, cTnT, CKMB, and BNP, which could be rescued by sh-miR-8055 expression in HCM cells, when exposed to LPS or not (Fig. 6C, D). Flow cytometry analysis suggested that silencing of circACSL1 inhibited cell apoptosis rate, but this effect was significantly abolished by knockdown of miR-8055 (Fig. 6E, F). It was further shown that silencing of miR-8055 rescued the reduction in MAPK14 level caused by circACSL1 knockdown (Fig. 6G). Consistently, the pro-inflammatory effects of circACSL1 on HCM were rescued by overexpression of miR-8055, and the induced expression of MAPK14 by circACSL1 was attenuated by miR-8055 overexpression (supplementary file 8). These data demonstrated that circACSL1 could act as a pro-inflammatory factor and regulate MAPK14 expression via the sponge activity of miR-8055 in cardiomyocytes.

### CircACSL1 aggravates myocardial inflammation and myocardial injury via miR-8055 /MAPK14 pathway

**Fig. 7:**
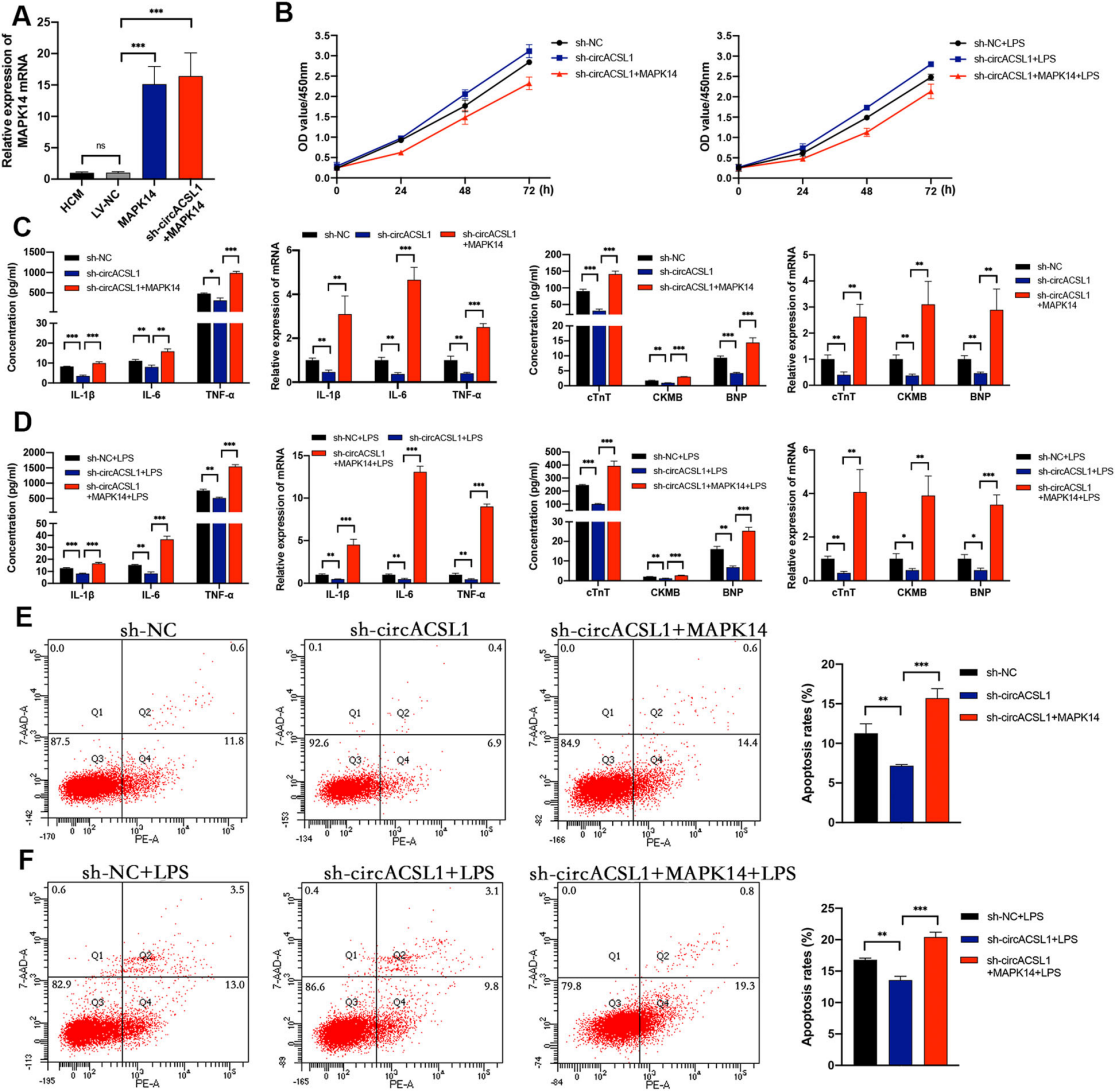
Overexpression of MAPK14 partly reverses the effects of sh-circACSL1. **A**–**F** The circACSL1-knockdown cell lines were co-transfected with MAPK14 overexpression vectors, which were compared with circACSL1-knockdown cells alone. **A** The overexpression efficiency of MAPK14 was detected by qRT-PCR. **B** Overexpression of MAPK14 abolished the suppressive effects of sh-circACSL1 on HCM cell proliferation. **C**, **D** Sh-circACSL1-induced downregulation of IL-1β, IL-6, TNF-α, cTnT, CKMB, and BNP were rescued by co-transfection with MAPK14 overexpression vectors and sh-circACSL1. **E**, **F** Sh-circACSL1-induced decrease of cell apoptosis was reversed by co-transfection with MAPK14 overexpression vectors and sh-circACSL1. Data are presented as mean ±SD (*n* = 3 biologically independent samples). *p < 0.05; **p < 0.01; ***p < 0.001 (Student’s *t* test).

**Fig. 8:**
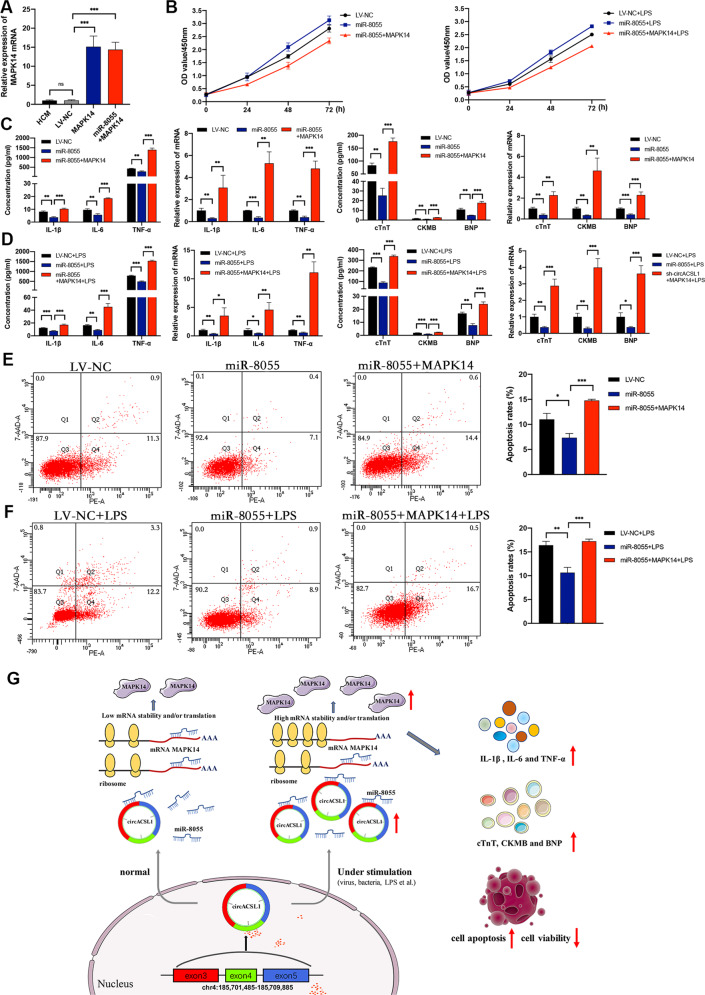
Overexpression of MAPK14 partly reverses the effects of miR-8055. **A**–**F** The miR-8055-overexpression cell lines were co-transfected with MAPK14 overexpression vectors, which were compared with miR-8055-overexpression cells alone. **A** The overexpression efficiencies of MAPK14 were detected by qRT-PCR. **B** Overexpression of MAPK14 abolished the suppressive effects of miR-8055 on HCM cell proliferation. **C**, **D** miR-8055 overexpression-induced downregulation of IL-1β, IL-6, TNF-α, cTnT, CKMB, and BNP were rescued by co-transfection with MAPK14 overexpression vectors and miR-8055 overexpression vectors. **E**, **F** miR-8055 overexpression-induced decrease of cell apoptosis was reversed by co-transfection with MAPK14 overexpression vectors and miR-8055 overexpression vectors. Data are presented as mean ± SD (n = 3 biologically independent samples). *p < 0.05; **p < 0.01; ***p < 0.001 (Student’s *t* test). **G** Graphical abstract. Graphical diagram of circACSL1 promoting myocardial inflammation and myocardial injury by sponging miR-8055 and regulating MAPK14 expression.

The above results have demonstrated that circACSL1 could exert pro-inflammatory effects and regulate MAPK14 expression by sponging miR-8055 in HCM cells. To further investigate whether circACSL1 exerts pro-inflammatory effects via miR-8055/MAPK14 pathway, we overexpressed MAPK14 in circACSL1-knockdown cell lines or miR-8055-overexpressed cell lines. As shown in Fig. 7, sh-circACSL1-mediated knockdown of circACSL1 showed a significant reduction in the expressions of pro-inflammatory cytokines (IL-1β, IL-6, and TNF-α), myocardial injury markers (cTnT, CKMB, and BNP), and cell apoptosis rate, which could be rescued by overexpression of MAPK14. Consistently, the effects of miR-8055 that alleviated myocardial inflammation and myocardial injury on HCM were significantly abolished by overexpression of MAPK14 (Fig. 8). These data demonstrated that circACSL1 aggravated myocardial inflammation and myocardial injury via miR-8055 /MAPK14 pathway.

## Discussion

MC is a common, potentially life-threatening inflammatory disease of the myocardium. It is one of the most challenging clinical problems as lacking specific diagnosis and effective therapy, while the exact etiology and pathogenesis remain poorly understood^1,6,11,44^. Emerging studies have suggested that circRNAs have a vital role in the onset and progression of cardiovascular diseases^20–22^, but only a handful of studies have focused on the biological roles of circRNA in myocardial injury and myocardial inflammation. Zhou J et al.^45^ have found that circ-HIPK2 facilitates autophagy to accelerate cellular apoptosis in H_2_O_2_-induced myocardial oxidative injury through the miR-485-5p/ATG101 pathway. Fan S et al.^46^ have also verified that interference of circRNA HIPK3 to alleviate LPS-induced cardiac dysfunction mice models and apoptosis in H9C2 cardiomyocytes as well. In our previous study^42^, we obtained a differential circRNA expression profile based on microarray analysis of peripheral blood leukocytes in children with MC. We then identified a particular circRNA, hsa_circ_0071542, which exhibited upregulated expression in MC. These findings led us to speculate that circ_0071542 might play a pro-inflammatory role in the progression of MC.

In the present study, we investigated the structural features and the mechanisms of circ_0071542, back spliced from ACSL1 pre-mRNA, in relation to MC. Herein, we termed it as circACSL1. Interestingly, even under RNase R treatment, circACSL1 demonstrated very high stability and integrity, similar to most other circRNAs. Therefore, we believe that circACSL1 has the potential to serve as a stable marker for the precise diagnosis of MC. From the clinical aspect, we determined that circACSL1 was significantly upregulated in the acute phase of MC and declined significantly during the recovery phase, suggesting it was related to the occurrence and progression of MC. Interestingly, the dynamic change of circACSL1 expression level was consistent with the trend of Hs-TnT and pro-NT BNP, which were well-defined myocardial injury and heart failure biomarkers in clinical diagnosis. Therefore, these data indicated that circACSL1 could serve as a clinical biomarker for diagnostic and prognostic evaluation of MC. The differential diagnosis between MC and DCM in acute phase has been challenging as the clinical manifestations (such as cardiac dilation, an increase of myocardial injury biomarkers, declination of left ventricular ejection fraction, and heart failure) were similar sometimes^5,43^, hence searching for a novel biomarker to differentiate MC from DCM for accurate diagnosis is urgently needed. Our research found that the expression of cirACSL1 in MC was dramatically higher than in DCM and controls, suggesting that circACSL1 might provide a new perspective for differential diagnosis of MC from DCM. ﻿However, whether circACSL1 can be used as a new biomarker for MC requires further verification by examining larger cohorts of samples.

Next, we used LPS-induced HCM inflammation model to investigate the pathobiological effects and mechanism of circACSL1 in vitro. Subsequent in vitro gain-of-function and loss-of-function experiments confirmed that circACSL1 could promote myocardial inflammation, aggravate the myocardial injury, suppress cell viability, and promote cell apoptosis. In addition, circACSL1 knockdown or overexpression had no effect on ACSL1 pre-mRNA expression, indicative of the independent role of circACSL1 in MC progression. The identified pro-inflammatory effects of circACSL1 in MC features it as a promising therapeutic target for MC treatment.

Generally, circRNAs are classified into three types: exonic circular RNA (ecircRNA), circular intronic RNA (ciRNA), and exon–intron circRNA (EIciRNA). EcircRNA mainly exists in the cytoplasm, serving as a “sponge” for microRNAs, whereas the other two circRNAs are confined to the nucleus owing to the presence of introns^13,14^. Some miRNAs are reported to exhibit aberrant expression levels in MC, and increasing evidence has highlighted the role of miRNAs in MC pathogenesis^47–49^. In this study, we discovered that circACSL1 was mainly located in the cytoplasm. CircACSL1 contains a conserved miR-8055 target site that was validated by luciferase reporter assay in HCM. In addition, we found that miR-8055 expression was significantly downregulated in MC, exhibiting anti-inflammatory effects. Subsequent analyses showed that miR-8055 suppressed MAPK14 expression, a mechanism reported herein for the first time. MAPK14 (p38α) is a serine/threonine kinase involved in inflammatory signaling cascades, and some studies have proven that it serves as a crucial modulator in MC progression. Marchant D et al.^38^ has proved that Bosentan, used for pulmonary arterial hypertension, could enhance viral load and MC severity through ETRA-mediated p38 MAPK activation and p38 MAPK is a desirable antiviral target in MC. Chen H et al.^40^ has found that long non-coding RNA MALAT1 regulates sepsis-induced cardiac inflammation and dysfunction via interaction with miR-125b and p38 MAPK/NFκB pathway. Under a wide variety of inflammatory stimuli, MAPK14 can activate downstream transcription factors or protein kinases, leading to the initiation of a series of inflammation reactions^35,36^. Our research showed that circACSL1 and miR-8055 could serve as upstream regulators to regulate MAPK14 expression.

Finally, we identified that cirACSL1 exerted pro-inflammatory effects on MC by sponging miR-8055 target sites and regulating MAPK14 expression through rescue assay. Therefore, we propose that targeting of the circACSL1/miR-8055/MAPK14 axis as a preventative strategy for MC treatment. However, we do not exclude the possibility of the involvement of other critical gene targets of circACSL1 besides MAPK14 that may play important roles in the progression of MC.

There were at least three limitations of the study, which must be mentioned here. First, all the MC patients in this study lacked myocardial biopsy, and they were diagnosed based on clinical criteria. Second, circACSL1 is less conserved between humans and mice with 80.9% sequence homology and we could not find a homologous sequence of human circACSL1 in rat or rabbit transcriptome. The same thing with circACSL1, there is no homologous sequence of human miR-8055 in mice, rat, or rabbit (supplementary file 9). Accordingly, we could not investigate the biological effects of circACSL1 and miR-8055 in rodent disease models. Finally, the identification of downstream substrates of MAPK14 signaling pathway and how ﻿MAPK14 regulates myocardial inflammation and myocardial injury through these downstream effectors remains for further investigation.

In summary, we discovered for the first time that circACSL1 was significantly upregulated in MC and dramatically aggravated HCM inflammation, leading to increased apoptotic cell death. The mechanism by which this occurred could be through competitive adsorption of miR-8055, thereby upregulating MAPK14 expression. Our results revealed, for the first time, that circACSL1 might represent a promising novel biomarker for the diagnosis of MC and drawing distinctive diagnostic lines from DCM. Moreover, these findings also provide new evidence for circRNAs-mediated pathomechanisms in MC onset and progression and offer a promising therapeutic target to broaden the treatment options.

## Supplementary information

The clinical characteristics of dilated cardiomyopathy (DCM)

The cell line authentication result (STR Profiling Report)

LPS-induced HCM inflammation model successfully

The overexpression sequences of circACSL1, MAPK14 and mutant circACSL1

The transfection efficiency of the overexpression or knockdown lentivirus vector

The sequences of primers used in qRT-PCR and biotinylated probe sequences used in RNA pull-down

The sequences of circACSL1 or the 3′-UTR of MAPK14 in dual-luciferase reporter assay

The pro-inflammatory effects of circACSL1 could be rescued by miR-8055 overexpression

The search results of homologous sequence of human miR-8055

The text summary for supplementary file

## Data Availability

All data generated or analyzed during this study are included in this published article (and its supplementary information files).
