# Supplementary material for: Circular RNA circACSL1 aggravated myocardial inflammation and myocardial injury by sponging miR-8055 and regulating MAPK14 expression
Source: Cell Death Dis. 2021 May 13;12(5):487. doi: 10.1038/s41419-021-03777-7 (PMC8119943; doi:10.1038/s41419-021-03777-7)
Supplement: Supplementary file 1 — The clinical characteristics of dilated cardiomyopathy (DCM) [file 41419_2021_3777_MOESM1_ESM.docx]

| **Table 1. Clinical ﻿characteristics of dilated cardiomyopathy (DCM). (n=25)** | |
| --- | --- |
| **Sex** | |
| Male | 10 |
| Female | 15 |
| ﻿**Age** (years) (median, P25, P75) | 1.42 (0.71, 7.42) |
| **Echo** | |
| **LVEDD Z-score** (mean ± SD) | 6.74 ± 2.69 |
| ﻿ **LVEF** (%) (mean ± SD) | 29.4 ± 7.88 |
| **NT-pro BNP** (%) (mean ± SD) | 12003 ± 11373 |
| **Echo, echocardiographic; LVEDD**, Left ventricular end-diastolic diameter; Z-score, after body surface area (BSA) correction, the distance from the average (normal range, 0±2); **LVEF**, Left ventricular ejection fraction (normal value >60%); **NT-pro BNP,** NT-pro brain natriuretic peptide, an index of heart failure (normal range, 0-450pg/ml). ﻿***p < 0.001 versus control. | |
