# Supplementary material for: Circular RNA circACSL1 aggravated myocardial inflammation and myocardial injury by sponging miR-8055 and regulating MAPK14 expression
Source: Cell Death Dis. 2021 May 13;12(5):487. doi: 10.1038/s41419-021-03777-7 (PMC8119943; doi:10.1038/s41419-021-03777-7)
Supplement: Supplementary file 2 — The cell line authentication result (STR Profiling Report) [file 41419_2021_3777_MOESM2_ESM.pdf]

# Cell Line Authentication – STR Profiling Report

Sample Type: Cell Line
Testing Type: STR

Sample code:

Table 1. Sample Code

| Customer's code | Company Code |
|---|---|
| 851 | 20210402-02 |

Sample Number:1

Sample Type: Cell line

Testing Type: STR

Sample From: Shanghai Zhong Qiao Xin Zhou Biotechnology Co.,Ltd.

中乔新舟

## Testing Method:

DNA was extracted by a commercial kit from CORNING (AP-EMN-BL-GDNA-250G). Twenty short tandem repeat (STR) loci plus the gender determining locus, Amelogenin, were amplified by six multiplex PCR and separated on ABI 3730XL Genetic Analyzer. The signals were then analyzed by the software GeneMapper.

## Data Interpretation:

Cell lines were authenticated using Short Tandem Repeat (STR) analysis as described in 2012 in ANSI Standard (ASN-0002) by the ATCC Standards Development Organization (SDO) and in Capes-Davis et al., Match criteria for human cell line authentication: Where do we draw the line? Int J Cancer. 2013;132(11):2510-9.

# Test Results:

## 1. Result

Table 2. Matching information on the cell lines

| Sample Code | Multi-allele | Cell line matched | Cell Bank | EV |
|---|---|---|---|---|
| 20210402-02 | YES | AC16 [Human hybrid] | EXPASY | 1.0 |

- Multi-allele means some STR contain more than two loci.

## 2. Sample Description

20210402-02:

A. The STR results showed that multiple alleles were found in this cell line, and no cross contamination of human cells was found in the cell line.

B. The DNA of the cell lines found to match the type of cell lines in a cell line retrieval, EXPASY database shows that cells called **AC16 [Human hybrid],** corresponding to the cell number **CVCL_4U18.**

中乔新舟

## 3. Genotyping Result

Table 3. STR and Amelogenin Genotyping Results of Cell line 20210402-02

| Loci | Sample information | | | Cell Bank information | | |
|---|---|---|---|---|---|---|
| | Sample name：851 | | | Cell line name：AC16 [Human hybrid] | | |
| | **Allele1** | **Allele2** | **Allele3** | **Allele1** | **Allele2** | **Allele3** |
| D5S818 | 9 | 11 | | 9 | 11 | |
| D13S317 | 12 | 13 | | 12 | 13 | |

| | | | | | | |
|---|---|---|---|---|---|---|
| D7S820 | 10 | 11 | 12 | 10 | 11 | 12 |
| D16S539 | 11 | 13 | | 11 | 13 | |
| VWA | 16 | 18 | | 16 | 18 | |
| TH01 | 7 | 8 | 9.3 | 7 | 8 | 9.3 |
| AMEL | X | X | | X | X | |
| TPOX | 11 | 11 | | 11 | 11 | |
| CSF1PO | 9 | 11 | 12 | 9 | 11 | 12 |
| D12S391 | 18 | 21 | | | | |
| FGA | 21 | 25 | | | | |
| D2S1338 | 20 | 23 | 26 | | | |
| D21S11 | 32.2 | 32.2 | | | | |
| D18S51 | 12 | 17 | | | | |
| D8S1179 | 14 | 14 | | | | |
| D3S1358 | 17 | 18 | | | | |
| D6S1043 | 14 | 17 | | | | |
| PENTAE | 7 | 7 | | | | |
| D19S433 | 15 | 15.2 | | | | |
| PENTAD | 9 | 9 | | | | |
| D1S1656 | 12 | 12 | | | | |

*The allele match algorithm compares the 8 core loci plus amelogenin only, even though alleles from all loci will be reported when available.*

中乔新舟

# Others:

1. Genotyping Strategy and Site Distribution

Attached Table. Experimental Strategy and Sites

| | Strategy 1 | Strategy 2 | Strategy 3 | Strategy 4 |
|---|---|---|---|---|
| 1 | D3S1358 | D8S1179 | D19S433 | AMEL |
| 2 | VWA | D21S11 | TH01 | D1S1656 |
| 3 | D7S820 | D16S539 | D13S317 | D5S818 |
| 4 | CSF1PO | D2S1338 | TPOX | D12S391 |
| 5 | PENTAE | PENTAD | D18S51 | FGA |
| 6 | | | D6S1043 | |

2. DSMZ tools was used to carry on the cell line comparison, which contains 2455 cell lines STR data from ATCC, DSMZ, JCRB, ECACC, GNE and RIKEN databases. If the cell is not included in the above cell library, users need to compared with other databases.

中乔新舟

中乔新舟
CELL RESEARCH

# Certificate of STR Analysis

Applied Biosystems
GeneMapper ID v3.2

20210402

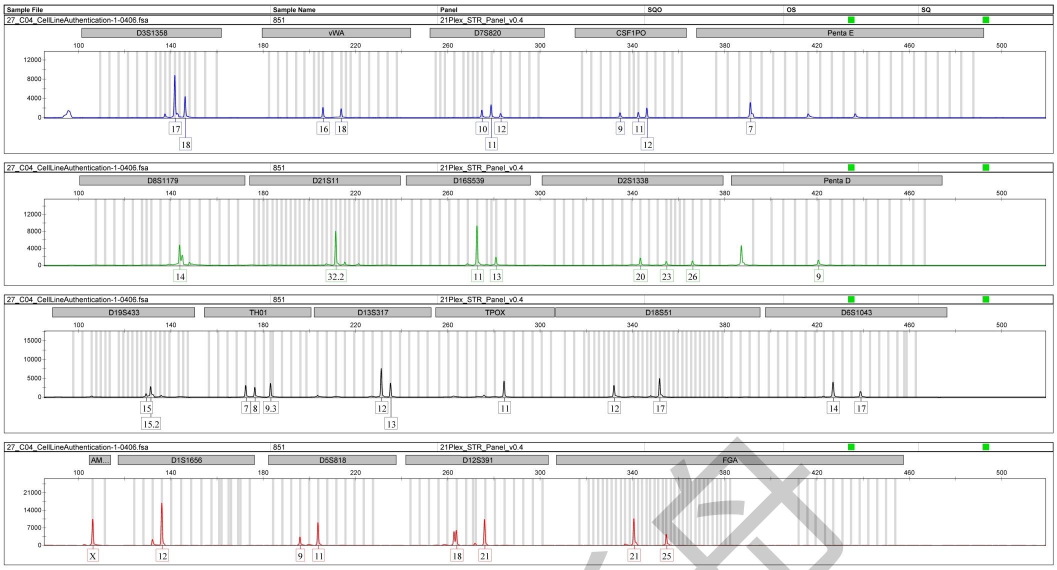


Wed Apr 07,2021 02:15PM, GMT+08:00 Printed by: gmid Page 1 of 1

中乔新舟

Report Date:
Apr., 2021
