# Supplementary figures and images for: Circular RNA circACSL1 aggravated myocardial inflammation and myocardial injury by sponging miR-8055 and regulating MAPK14 expression
Source: Cell Death Dis. 2021 May 13;12(5):487. doi: 10.1038/s41419-021-03777-7 (PMC8119943; doi:10.1038/s41419-021-03777-7)

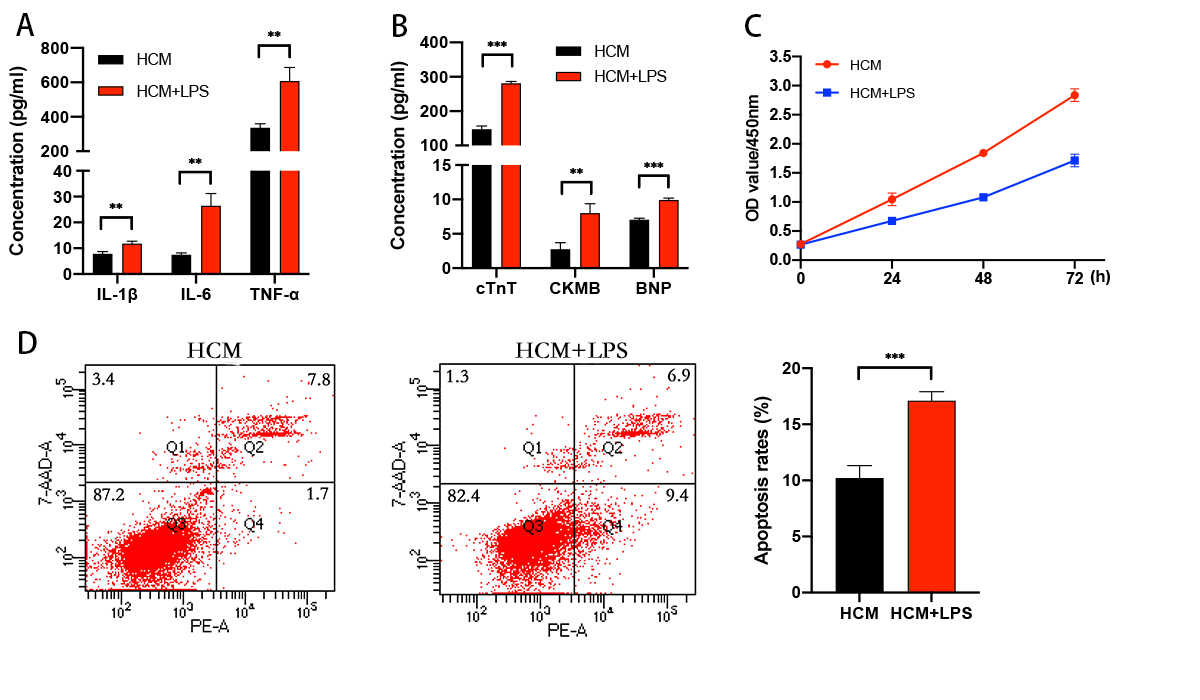

Supplement: Supplementary file 3 — LPS-induced HCM inflammation model successfully [file 41419_2021_3777_MOESM3_ESM.png]

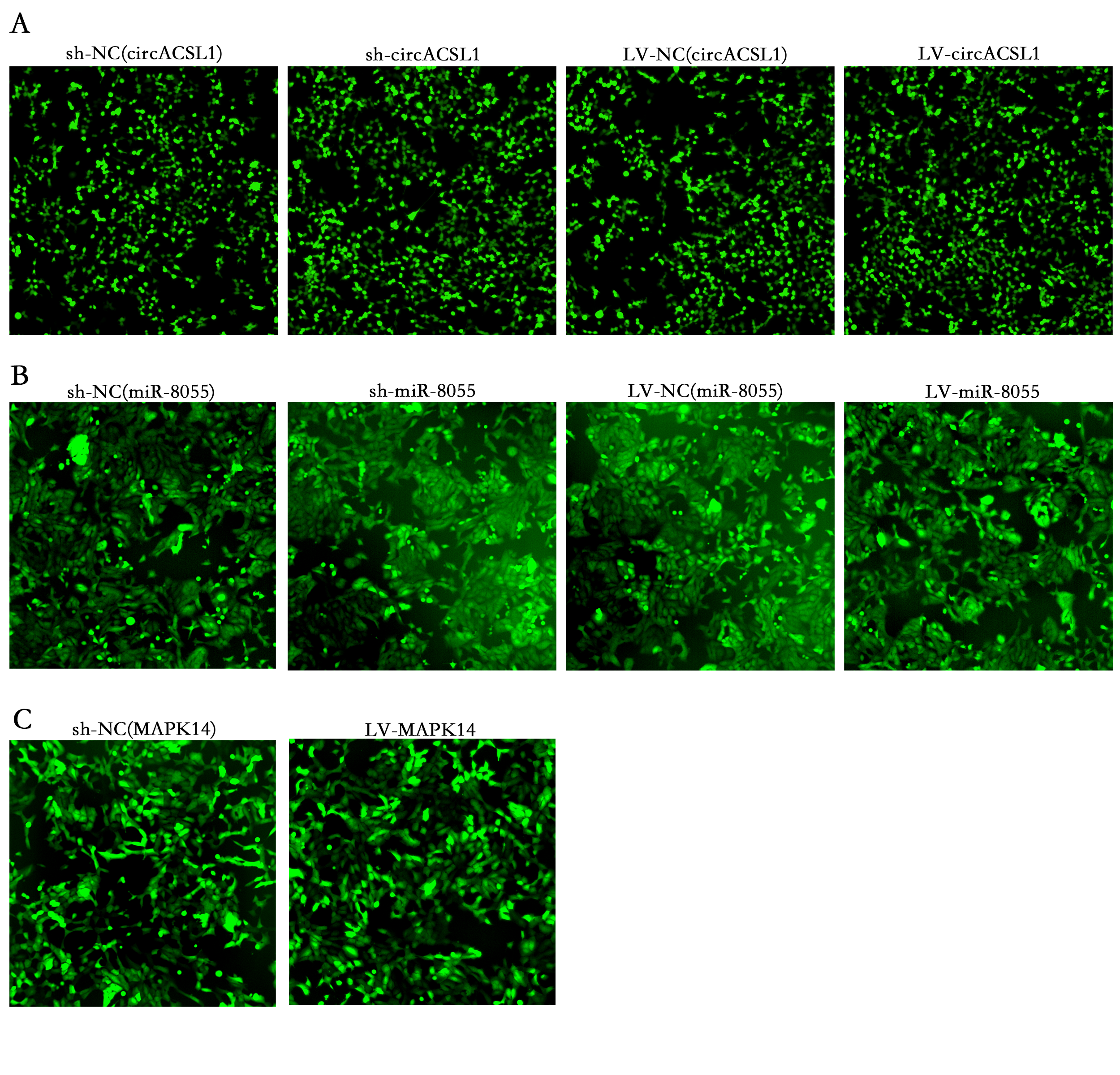

Supplement: Supplementary file 5 — The transfection efficiency of the overexpression or knockdown lentivirus vector [file 41419_2021_3777_MOESM5_ESM.png]

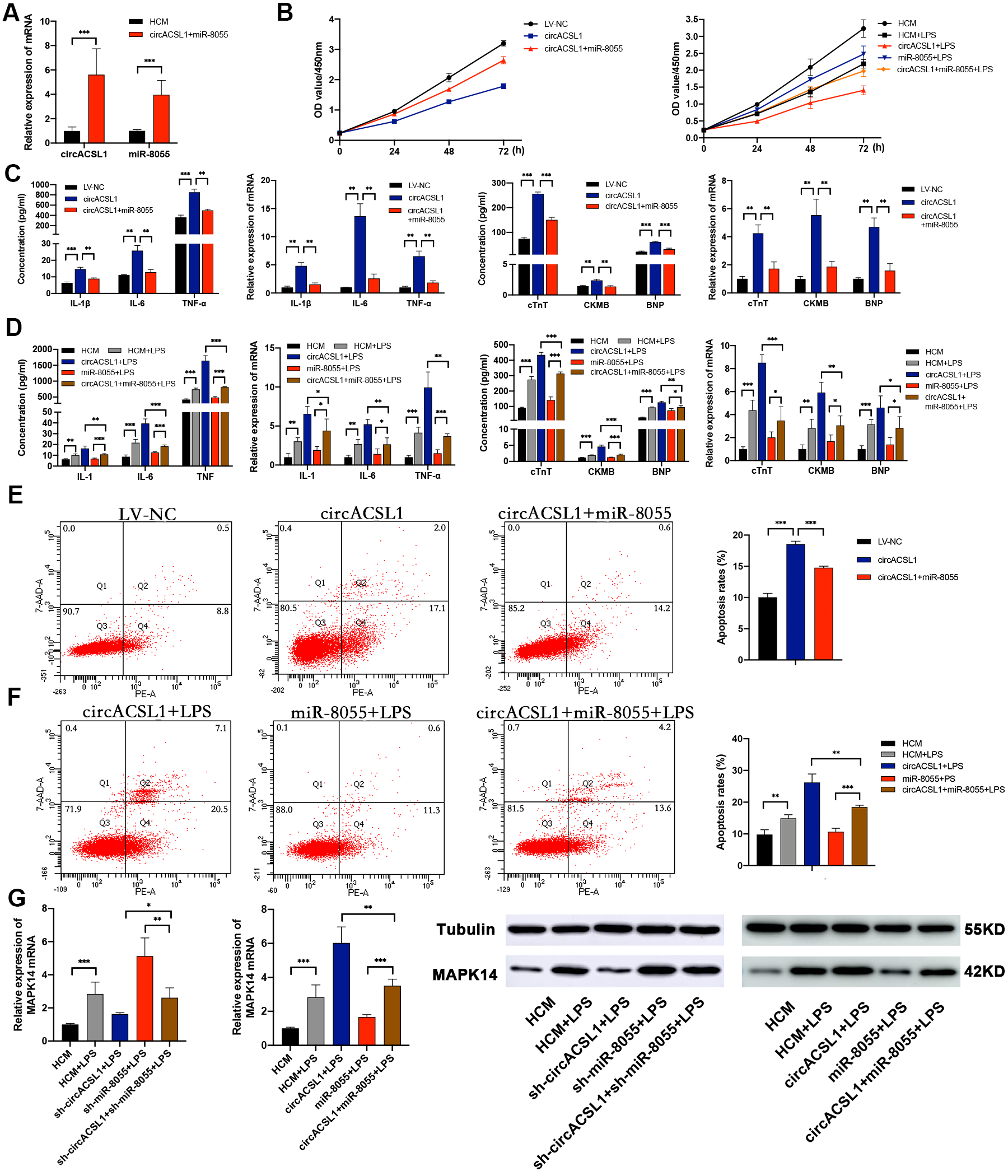

Supplement: Supplementary file 8 — The pro-inflammatory effects of circACSL1 could be rescued by miR-8055 overexpression [file 41419_2021_3777_MOESM8_ESM.png]
