# Supplementary material for: Circular RNA circACSL1 aggravated myocardial inflammation and myocardial injury by sponging miR-8055 and regulating MAPK14 expression
Source: Cell Death Dis. 2021 May 13;12(5):487. doi: 10.1038/s41419-021-03777-7 (PMC8119943; doi:10.1038/s41419-021-03777-7)
Supplement: Supplementary file 4 — The overexpression sequences of circACSL1, MAPK14 and mutant circACSL1 [file 41419_2021_3777_MOESM4_ESM.docx]

**Overexpression sequences of circACSL1 (hsa_circ_0071542)**

GAACTGTCAATGTGTGTAGTGGTGGCAGTTTGTGGCTCTTCTCTTGCTCTCTTTGGCCTTATCCGGCAGGACCAGCACTTGTAGGTCTGTAAACCTGTTTTCTCTCTTCAGGCATCTTTCTGACTTAGCCGTGGTTCCCCGAAGCCTGTGTTTACCTCCAGGCGAATGATTGGTTTACATCAGCACATCTTGCACTGCAGTCTTTCAGCACTCACTCACTGGGCAGCTGTTCCGCAGGTGGCCGCCTAGAGGGGTGCGGATACCACAGGGGCATATTAGACTTTATATTTAATTTTTAAGCATTTTATTTTTTAATGGACAAATACAAATTATGTGTATTTATGGTGTATAATAGGAACGAGTTCAGGACATCTATTTTTAATAAAAATGGTGACCATGTTCACTATTAGTTGGTGACTGTAGTTAATAACAATGTACCATATACCTGAAATTACTAAGAGAATAGATTTTAAGTGTTCTCACTACAGATAAGTGACAAGAATGTAAAGGAATGGATATGTTAATTAGCTTGATTTAGCCATTCCACAGTGCATAGATGTTTCACTGCCTTCTAACCTGCCCCCCTTTCTTTTTTGATAGGGTAGTGGTGGTGCACGAAGATCCGCACTACTTGACAGCGACGAGCCCTTGGTGTATTTCTATGATGATGTCACAACATTATACGAAGGTTTCCAGAGGGGAATACAGGTGTCAAATAATGGCCCTTGTTTAGGCTCTCGGAAACCAGACCAACCCTATGAATGGCTTTCATATAAACAGGTTGCAGAATTGTCGGAGTGCATAGGCTCAGCACTGATCCAGAAGGGCTTCAAGACTGCCCCAGATCAGTTCATTGGCATCTTTGCTCAAAATAGACCTGAGGTATGAACCAGTAGGTACTTGCTATGCATGATTTTGAGAATTCTCAGATATAAGAGCCTAACTTCTGCTAGAGACCAGATTTTGTAGTCTGATTTCTACTAAAAGTATAGCTTTACGTTACACCTTAGAAAGTTACACATATGAAAACCTCAACCAGGGCCGCTGGGCACAGTGGCTCACGCCTGTAATCCCAGCACTTTGTGAGGCCAAGGCGGGTGGGTCACCAGAGGTCGGGAGTTCCAGACCAGCCTGACCAACATGGAGAAACCCCATCTCTACTAAAAATACAAAATTAGCCAGGCCTGGTGGCACACATCTGTAATCCCAGCTACTCGGGAGGCTGAGGCAGGAGAATCGCTTGAATCCGGGAGGCAGAGGTTGCGGTGAGCCAAGATTGTGCCATTGCACTCCAGCCTGGGCAACAAGAGCGAAACTCCGTCTCAAAAAAAAAAAAAAAGAAAGGAAAACCTGAACCAGGGCCTTTTAATCGCCTTTAAGAGAAAAAAGAAATCACTTTTTTGGGGGGATTATTTTAGTTTTTTTAAACCAGGTTCCCAAATAAAATCCTTAGAACAGATAACTAACAAAGGAGTCTTTTTA

**Overexpression sequences of MAPK14**

GGCTTTTTTGTTAGACGAAGCTTGGGCTGCAGGTCGACTCTAGAGGATCCCCGGGTACCGGTCGCCACCATGTCTCAGGAGAGGCCCACGTTCTACCGGCAGGAGCTGAACAAGACAATCTGGGAGGTGCCCGAGCGTTACCAGAACCTGTCTCCAGTGGGCTCTGGCGCCTATGGCTCTGTGTGTGCTGCTTTTGACACAAAAACGGGGTTACGTGTGGCAGTGAAGAAGCTCTCCAGACCATTTCAGTCCATCATTCATGCGAAAAGAACCTACAGAGAACTGCGGTTACTTAAACATATGAAACATGAAAATGTGATTGGTCTGTTGGACGTTTTTACACCTGCAAGGTCTCTGGAGGAATTCAATGATGTGTATCTGGTGACCCATCTCATGGGGGCAGATCTGAACAACATTGTGAAATGTCAGAAGCTTACAGATGACCATGTTCAGTTCCTTATCTACCAAATTCTCCGAGGTCTAAAGTATATACATTCAGCTGACATAATTCACAGGGACCTAAAACCTAGTAATCTAGCTGTGAATGAAGACTGTGAGCTGAAGATTCTGGATTTTGGACTGGCTCGGCACACAGATGATGAAATGACAGGCTACGTGGCCACTAGGTGGTACAGGGCTCCTGAGATCATGCTGAACTGGATGCATTACAACCAGACAGTTGATATTTGGTCAGTGGGATGCATAATGGCCGAGCTGTTGACTGGAAGAACATTGTTTCCTGGTACAGACCATATTAACCAGCTTCAGCAGATTATGCGTCTGACAGGAACACCCCCCGCTTATCTCATTAACAGGATGCCAAGCCATGAGGCAAGAAACTATATTCAGTCTTTGACTCAGATGCCGAAGATGAACTTTGCGAATGTATTTATTGGTGCCAATCCCCTGGCTGTCGACTTGCTGGAGAAGATGCTTGTATTGGACTCAGATAAGAGAATTACAGCGGCCCAAGCCCTTGCACATGCCTACTTTGCTCAGTACCACGATCCTGATGATGAACCAGTGGCCGATCCTTATGATCAGTCCTTTGAAAGCAGGGACCTCCTTATAGATGAGTGGAAAAGCCTGACCTATGATGAAGTCATCAGCTTTGTGCCACCACCCCTTGACCAAGAAGAGATGGAGTCCGGTATGGACTACAAGGATGACGATGACAAGGATTACAAAGACGACGATGATAAGGACTATAAGGATGATGACGACAAATGAGCTAGCCTGTGGAATGTGTGTCAGTTAGGGTGTGGAAAGTCCCCAGGCTCCCCAGCAGGCAGAAGTATGCAAAGCATGCATCTCAATTAGTCAGCAACCAGGTGTG

**Sequences of mutant circACSL1**

CTAAAATTATTCGTTCATGGCTTGAGTTCTAAAATTAAACTATGTGGAGTCATGTCCACCCGCACAATGCATCTTTATGTGAAACTTGCTAGAGTTTTTGTTTTCCTTCTATGTAAAAGTCCAGTTGGGAAGCTTTATTTCTGATAGATTAAATGGTATAGGTCTTTCAGTTTTCTCTTCATTTCTGACAACTGAACTGCTCTCGCCTTGAACCTGTTTTGGCGGTACCAGGGTAGTGGTGGTGCACGAAGATCCGCACTACTTGACAGCGACGAGCCCTTGGTGTATTTCTATGATGATGTCACAACATTATACGAAGGTTTCCAGAGGGGAATACAGGTGTCAAATAATGGCCCTTGTTTAGGCTCTCGGAAACCAGACCAACCCTATGAATGGCTTTCATATAAACAGGTTGCAGAATTGTCGGAGTGCATAGGCTCAGCACTGATCCAGAAGGGCTTCAAGACTGCCCCAGATCAGTTCATTGGCATCTTGTAGACCCATAGACCTGAGGTGGATCCGCCAAAACAGGTTCAAGGCGAGAGCAGTTCAGTTGTCAGAAATGAAGAGAAAACTGAAAGACCTATACCATTTAATCTATCAGAAATAAAGCTTCCCAACTGGACTTTTACATAGAAGGAAAACAAAAACTCTAGCAAGTTTCACATAAAGATGCATTGTGCGGTTGGAC

GTAGACCC are the mutant sites (in miR-8055 bind sites).
