# Supplementary material for: Circular RNA circACSL1 aggravated myocardial inflammation and myocardial injury by sponging miR-8055 and regulating MAPK14 expression
Source: Cell Death Dis. 2021 May 13;12(5):487. doi: 10.1038/s41419-021-03777-7 (PMC8119943; doi:10.1038/s41419-021-03777-7)
Supplement: Supplementary file 6 — The sequences of primers used in qRT-PCR and biotinylated probe sequences used in RNA pull-down [file 41419_2021_3777_MOESM6_ESM.docx]

| **Primer name** | **Primer sequence (5’-3’)** |
| --- | --- |
| hsa_circ_0071542 (circACSL1) | F: 5’ GCAGAATTGTCGGAGTGCAT 3’  R: 5’ GCACCACCACTACCCTCAG 3’ |
| hsa_miR-8055 | F: 5’ GAGCACATGAGCAGACGGAAA 3’ |
| ACSL1 | F: 5’ GGAAGAGCCAACAGACGGAA 3’  R: 5’ CCTTTGGGGTTGCCTGTAGT 3’ |
| MAPK14 | F: 5’ CTACCGGCAGGAGCTGAACAA 3’  R: 5’ AATGATGGACTGAAATGGTCTGGAG 3’ |
| IL-1β | F: 5’ CTTCTGGGAAACTCACGGCA 3’  R: 5’ AGCACACCCAGTAGTCTTGC 3’ |
| IL-6 | F: 5’ TGAGGAGACTTGCCTGGTGAA 3’  R: 5’ CAGCTCTGGCTTGTTCCTCAC 3’ |
| TNF-α | F: 5’ GGGCAGGTCTACTTTGGGAT 3’  R: 5’ AGGTTGAGGGTGTCTGAAGG 3’ |
| cTnT | F: 5’CCAGAAAGTCTCCAAGACCCG 3’  R: 5’TCCCCATTTCCAAACAGGAGC 3’ |
| CKMB | F: 5’GCCATTCGGTAACACCCACA 3’  R: 5’TGTTTGCTGAGGTCGGGGTA 3’ |
| BNP | F: 5’ TCAGCCTCGGACTTGGAAAC 3’  R: 5’CTTCCAGACACCTGTGGGAC 3’ |

**Primer sequences used in qRT-PCR**

**Biotinylated probe sequences used in RNA pull-down**

| **Primer name** | **Primer sequence (5’-3’)** |
| --- | --- |
| circACSL1 | GTGCACCACCACTACCCTCAGGTC |
| Circ-NC | TCAAAATAGACCTGAGGGTAGTGGTGGTGCAC |
| hsa_miR-8055 | CUUUGAGCACAUGAGCAGACGGA |
| miR-NC | CTTTGAGCACATGAGCAGACGGA |
