# Supplementary material for: Circular RNA circACSL1 aggravated myocardial inflammation and myocardial injury by sponging miR-8055 and regulating MAPK14 expression
Source: Cell Death Dis. 2021 May 13;12(5):487. doi: 10.1038/s41419-021-03777-7 (PMC8119943; doi:10.1038/s41419-021-03777-7)
Supplement: Supplementary file 7 — The sequences of circACSL1 or the 3′-UTR of MAPK14 in dual-luciferase reporter assay [file 41419_2021_3777_MOESM7_ESM.docx]

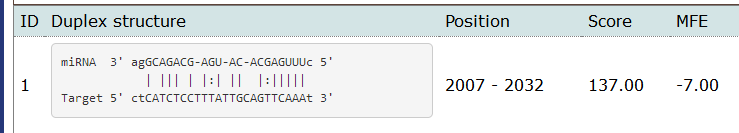


**3’UTR of MAPK14 (wide type)**

AGGAAGGAGGCAGACTGATGGCGATTCCCTCTCACCCGGGACTCTCCCCCTTTCAAGGAAAGTGAACCTTTAAAGTAAAGGCCTCATCTCCTTTATTGCAGTTCAAATCCTCACCATCCACAGCAAGATGAATTTTATCAGCCATGTTTGGTTGTAAATGCTCGTGTGATTTCCTACAGAAATACTGCTCTGAATATTTTGTAATAA

**3’UTR of MAPK14 (mutant type)**

AGGAAGGAGGCAGACTGATGGCGATTCCCTCTCACCCGGGACTCTCCCCCTTTCAAGGAAAGTGAACCTTTAAAGTAAAGGCCTCATCTCCTTTATTGCATGGACCCTCCTCACCATCCACAGCAAGATGAATTTTATCAGCCATGTTTGGTTGTAAATGCTCGTGTGATTTCCTACAGAAATACTGCTCTGAATATTTTGTAATAA

**circACSL1(wide type)**

GGTAGTGGTGGTGCACGAAGATCCGCACTACTTGACAGCGACGAGCCCTTGGTGTATTTCTATGATGATGTCACAACATTATACGAAGGTTTCCAGAGGGGAATACAGGTGTCAAATAATGGCCCTTGTTTAGGCTCTCGGAAACCAGACCAACCCTATGAATGGCTTTCATATAAACAGGTTGCAGAATTGTCGGAGTGCATAGGCTCAGCACTGATCCAGAAGGGCTTCAAGACTGCCCCAGATCAGTTCATTGGCATCTTTGCTCAAAATAGACCTGAG

**circACSL1(mutant type)**

GGTAGTGGTGGTGCACGAAGATCCGCACTACTTGACAGCGACGAGCCCTTGGTGTATTTCTATGATGATGTCACAACATTATACGAAGGTTTCCAGAGGGGAATACAGGTGTCAAATAATGGCCCTTGTTTAGGCTCTCGGAAACCAGACCAACCCTATGAATGGCTTTCATATAAACAGGTTGCAGAATTGTCGGAGTGCATAGGCTCAGCACTGATCCAGAAGGGCTTCAAGACTGCCCCAGATCAGTTCATTGGCATCTTGTAGACCCATAGACCTGAG

**Red base represents the binding sites with the seed area of miR-8055.**
