# Supplementary material for: Circular RNA circACSL1 aggravated myocardial inflammation and myocardial injury by sponging miR-8055 and regulating MAPK14 expression
Source: Cell Death Dis. 2021 May 13;12(5):487. doi: 10.1038/s41419-021-03777-7 (PMC8119943; doi:10.1038/s41419-021-03777-7)
Supplement: Supplementary file 9 — The search results of homologous sequence of human miR-8055 [file 41419_2021_3777_MOESM9_ESM.docx]

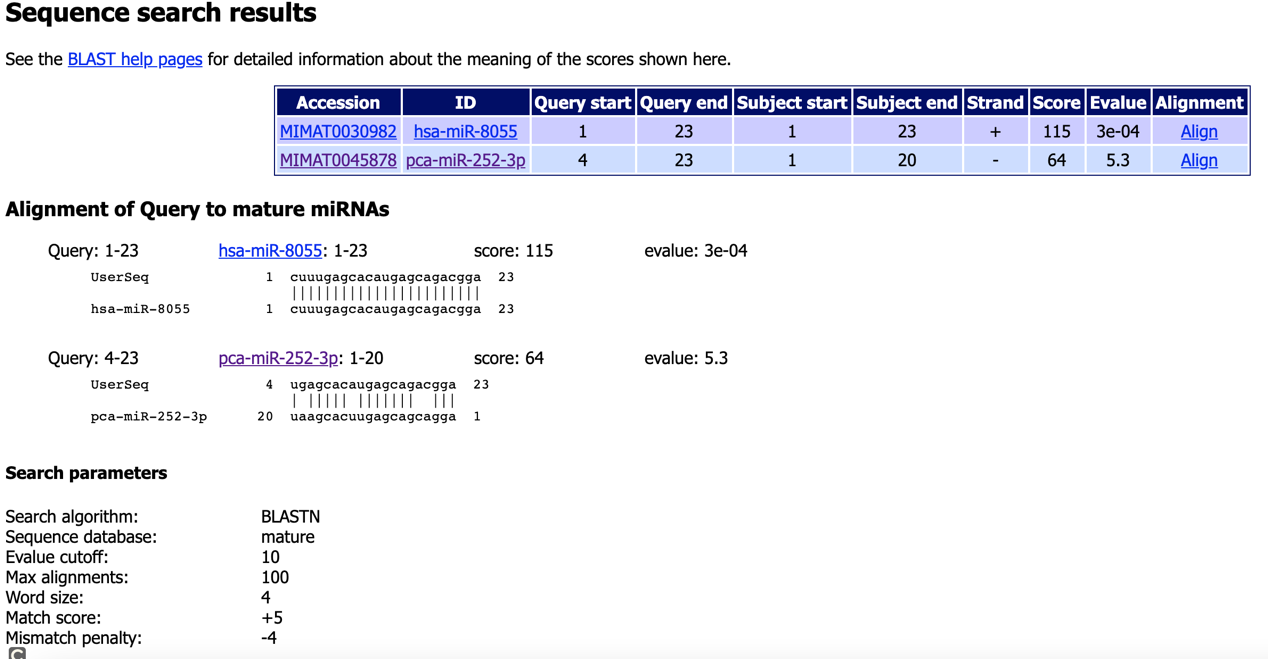


The sequence search results show that there is no homologous sequence of human miR-8055 in mice, rat or rabbit.
