# Supplementary material for: Circular RNA circACSL1 aggravated myocardial inflammation and myocardial injury by sponging miR-8055 and regulating MAPK14 expression
Source: Cell Death Dis. 2021 May 13;12(5):487. doi: 10.1038/s41419-021-03777-7 (PMC8119943; doi:10.1038/s41419-021-03777-7)
Supplement: Supplementary file 10 — The text summary for supplementary file [file 41419_2021_3777_MOESM10_ESM.docx]

**Supplementary file 1** showed the clinical characteristics of dilated cardiomyopathy (DCM).

**Supplementary file 2 s**howed the cell line authentication result (STR Profiling Report).

**Supplementary file 3** showed that LPS induced HCM inflammation model successfully. (A) The expression level of IL-1β, IL-6 and TNF-α detected by ELISA. (B) The expression level of cTnT, CKMB and BNP detected by ELISA. (C) Growth curves of HCM with or without LPS stimulation were measured by the CCK-8 assay. (D) ﻿Cell apoptosis was measured with annexin V-FITC/7-AAD double staining using ﻿flow cytometry apoptosis assay.

**Supplementary file 4** showed the overexpression sequences of circACSL1, MAPK14 and mutant circACSL1.

**Supplementary file 5** showed the transfection efficiency of the overexpression or knockdown lentivirus vector of circACSL1, mir-8055 and MAPK14 by detecting GFP expression using fluorescence microscope.

**Supplementary file 6** showed the sequences of primers (circACSL1, miR-8055, ACSL1, MAPK14, IL-1β, IL-6, TNF-α, cTnT, CKMB and BNP) used in qRT-PCR and biotinylated probe sequences used in RNA pull-down.

**Supplementary file 7** showed the sequences of circACSL1 or the 3′-UTR of MAPK14, including wild-type (circACSL1-Wt, MAPK14-Wt) or mutant miR-8055 binding sites (circACSL1-Mut, MAPK14-Mut) in dual-luciferase reporter assay.

**Supplementary file 8** showed the pro-inflammatory effects of cirACSL1 could be rescued by miR-8055 overexpression. (A-G) HCM cells were co-transfected with circACSL1 and miR-8055 overexpression vectors, which were compared with circACSL1 overexpression alone or miR-8055 overexpression alone. (A) The overexpression efficiencies of circACSL1 and miR-8055 in HCM cells were detected by qRT-PCR. (B) HCM proliferation curves were measured by CCK-8 assay. (C-D) The expression levels of IL-1β, IL-6, TNF-α, cTnT, CKMB, and BNP were estimated by qRT-PCR and ELISA. (E-F) Cell apoptosis was measured with annexin V-FITC/7-AAD double staining using ﻿flow cytometry apoptosis assay. (G) qRT-PCR and WB results showed the mRNA and protein levels of MAPK14. ﻿Data are presented as mean ± SD (n = 3 biologically independent samples). *P < 0.05; **P < 0.01; ***P < 0.001 (Student’s t-test).

**Supplementary file 9** showed the search results of homologous sequence of human miR-8055.
